# Influence of vintage, geographic location and cultivar on the structure of microbial communities associated with the grapevine rhizosphere in vineyards of San Juan Province, Argentina

**DOI:** 10.1371/journal.pone.0243848

**Published:** 2020-12-14

**Authors:** Mónica Oyuela Aguilar, Alex Gobbi, Patrick D. Browne, Lea Ellegaard-Jensen, Lars Hestbjerg Hansen, Liliana Semorile, Mariano Pistorio

**Affiliations:** 1 Instituto de Biotecnología y Biología Molecular (IBBM), CCT-La Plata, CONICET, Dto de Cs. Biológicas, Fac. Cs. Exactas, UNLP, La Plata, Argentina; 2 Section of Microbial Ecology and Biotechnology, Department of Plant and Environmental Sciences, University of Copenhagen, Frederiksberg, Denmark; 3 Department of Environmental Science, Aarhus University, Roskilde, Denmark; 4 Laboratorio de Microbiología Molecular, Departamento de Ciencia y Tecnología, Instituto de Microbiología Básica y Aplicada, Universidad de Nacional de Quilmes (UNQ), Bernal, Buenos Aires, Argentina; Institute for Sustainable Plant Protection, C.N.R., ITALY

## Abstract

Soil microbiomes, as a primary reservoir for plant colonizing fungi and bacteria, play a major role in determining plant productivity and preventing invasion by pathogenic microorganisms. The use of 16S rRNA and ITS high-throughput amplicon sequencing for analysis of complex microbial communities have increased dramatically in recent years, establishing links between wine specificity and, environmental and viticultural factors, which are framed into the elusive *terroir* concept. Given the diverse and complex role these factors play on microbial soil structuring of agricultural crops, the main aim of this study is to evaluate how external factors, such as vintage, vineyard location, cultivar and soil characteristics, may affect the diversity of the microbial communities present. Additionally, we aim to compare the influence these factors have on the structuring of bacterial and fungal populations associated with Malbec grapevine rhizosphere with that of the more widespread Cabernet Sauvignon grapevine cultivar. Samples were taken from Malbec and Cabernet Sauvignon cultivars from two different vineyards in the San Juan Province of Argentina. Total DNA extracts from the rhizosphere soil samples were sequenced using Illumina’s Miseq technology, targeting the V3-V4 hypervariable 16S rRNA region in prokaryotes and the ITS1 region in yeasts. The major bacterial taxa identified were *Proteobacteria*, *Bacteroidetes* and *Firmicutes*, while the major fungal taxa were *Ascomycetes*, *Basidiomycetes*, *Mortierellomycetes* and a low percentage of *Glomeromycetes*. Significant differences in microbial community composition were found between vintages and vineyard locations, whose soils showed variances in pH, organic matter, and content of carbon, nitrogen, and absorbable phosphorus.

## Introduction

Plant-microbe interactions are both dynamic and complex in terms of beneficial and deleterious associations, which play a key role in plant growth, tolerance against stresses, nutrition, productivity and product quality [1]. It has been suggested that vineyard soils impart a unique quality to the grapes and wine due to physiological responses of vines to soil type, topography and climatic conditions, in addition to the agricultural practices implemented [2, 3]. Plant roots are colonized by a subset of organisms from the soil microbiome creating rhizosphere and endosphere communities enriched with specific species [4].

In previous studies, distinct bacterial and fungal communities were found to be associated with vineyard soils, roots, trunks [5, 6], leaves [7], grapes, flowers and grape musts [1, 3, 8]. However, the effects of these communities on grape metabolism are not yet well known. Differences between microbial populations in the grapevine phyllosphere have been attributed to grapevine genotype [9], which in interaction with the geographical area, climatic factors, vine and grape health, lead to the concept of microbial *terroir* [10]. This concept has been reinforced at a phenotype-metabolome level by works from Knight et al. [11], Bokulich et al. [12] and Belda et al. [13]. Zarraonaindia et al. [3] have shown evidence that structure of microbial communities may be more similar between grapes and must samples, suggesting that communities present on grapes, prior to fermentation, remains relatively stable. These authors further showed that soil serves as a key source of grapevine-associated microbes, and that the edaphic factor plus the particular vineyard characteristics may influence the pre-harvest native root microbiome.

Vega-Avila et al. [14] found that certain vineyard agricultural practices altering the availability of organic matter and soil nutrients affected the structure of the soil microbial community. Additionally, Marasco et al. [15] showed that changes in the rootstock genotype could affect the grapevine root systems and its associated microbiome. Grapevine-associated microbial communities may play specific roles in the productivity and disease resistance of their host plant. Also, microbial communities have the potential to influence the wine organoleptic properties, contributing to a regional *terroir* [16, 17]. The knowledge of factors influencing the structure of these microbiomes may provide insights into vineyard agricultural practices to shape and craft particular wine properties.

Worldwide, Argentina occupied eighth place in grapevine surface and fifth place in wine production in 2018 according to the OIV [18]. The main producing areas are Mendoza and San Juan Provinces [19]. So far, there have been few studies on the diversity of microbial communities associated with Argentine vineyards performed with 16S rRNA gene high-throughput amplicon sequencing [14]. Previous studies have shown that nutrient availability has an important effect on soil microbial composition [20, 21]. However, much is still to be revealed regarding how different soil nutrients affect the composition of microbial communities, plant health and plant-microbe interactions. Additionally, the constantly changing external conditions, e.g. agricultural practices, topography, climate, etc., have been shown to influence the restructuring of microbiomes and consequently, soil fertility, plant health and crop quality. Although the taxonomic diversity analysis by itself is not informative regarding the metabolic functioning of microbial communities, a shift in its composition is considered a clear sign of community restructuring [22], which may reflect a functional modification [23] and impact the overall plant microbiome, producing an effect on the resulting wine [24].

In this study, we characterized the bacterial and fungal communities associated with the rhizosphere of two different grapevine cultivars, Malbec and Cabernet Sauvignon. Amplicons of two gene markers, 16S rRNA and ITS1, were used in order to identify if soil, cultivar and/or different vintages can be strong influential factors in shaping the microbial niches of the rhizosphere microbial communities present in vineyards from the Ullum Valley of San Juan, Argentina. With this purpose, we examined the soil physical and chemical composition of two Malbec and two Cabernet Sauvignon cultivars located in two different vineyards, seven days prior to the harvest periods of 2015, 2016 and 2017. To our knowledge, Vega Avila et al. [14] is the first and only *microbial terroir* study so far, in any Argentinian wine-region, where bacterial metabarcoding was the only analyzed data. Therefore, this research’s outcome will allow a better understanding of the bacterial and fungal ecology of vineyards in Argentina, prompting an improvement of this country’s agricultural practices, health of vines and wine quality.

## Materials and methods

### Sample collection

The permit to work on site came directly from the Ansilta vineyard and winery owner, H.J.V. Vignoli. Soil and root samples came from two vineyards located in the Ullum Valley of San Juan Province, Argentina, 6 km from each other: Finca Norte (FN) (S 31° 27.114’W 068° 42.523’, 780 m above sea level) and Finca Arriba (FA) (S 31° 28.407’W 068° 45.486’, 800 m above sea level). Grapevines of Malbec (MA) and Cabernet Sauvignon (CS) cultivars were grown at both vineyards, in which a total of four plots were sampled. The vines were 26 years old with 1.5 m distances between vineyard rows, and 1 m distance in between the vines that were confined to each row. None of the grapevine cultivars were grafted and all where trained in a pergola system, with the exception of the Malbec vineyard from Finca Norte (FNMA), which was trained in a vertical shoot position. Also, according to the information provided, all sampled plots were subjected to similar conventional agricultural practices, e.g. same furrow irrigation practice, machinery and crop management.

Sampling was done a week before the harvest period in the years 2015, 2016 and 2017. Soil and root samples were collected in each vineyard at 30 cm depth, and at 20–30 cm distance from vine trunks. The study included 9 sampled vines per plot, covering a 49 m^2^ area, placed at least 7 m away from the plot edge. Samples were kept in sterile containers, transported on ice, and stored at −20°C until analysis. The nine samples were pooled in three biological replicates (S1 Fig).

### Determination of soil physicochemical characteristics

Soil samples were sent to the Centro Nacional de Investigaciones Agropecuarias (CNIA-INTA) (Buenos Aires, Argentina) for physicochemical analysis. Soil samples were first dried at 40°C, and then dissociated and sieved, according to pre-treatment soil standards (ISO 11464—*Soil quality*. *Pretreatment of samples for physico-chemical analysis*) [25].

The pH was measured with the potentiometric method with distilled water at a relationship of 1: 2.5 (ISO 10694—*Soil quality determination of pH* norms). The organic carbon was evaluated by a strong oxidizing microscale mixture [26], organic material by a mass loss calcination method [27], and organic nitrogen through the Kjeldahl method [28]. To determine soil phosphorus content, the method by Bray & Kurtz (1945) [29] was applied, and soil texture was analyzed according to Kilmer et al. (1949) [30]. To establish the soil type in each vineyard, acquired lime, sand and clay content were determined, using the USDA soil texture diagram.

### Rhizosphere sample preparation and DNA extraction

The rhizosphere soil was carefully removed from the roots and collected with a sterile metal spoon; scalpels were used to carefully rub off the adhering soil. Rhizosphere soil samples were later sieved to eliminate remaining roots and plant debris (pore size 0.5 mm). DNA extractions were performed from 0.4 g of rhizosphere soil, using the FastDNA Spin Kit for Soil (MP Biomedicals, LLC, Solon, OH, USA), following the supplier’s instructions. The extracted DNA was quantified with a Qubit® 2.0 Fluorometer (Thermo Scientific™). DNA purity was assessed using a Nanodrop spectrophotometer, and determining the absorbance ratios 260/280 nm, and 260/230 nm.

### Library preparation and sequencing

The bacterial diversity was analyzed by amplifying the hypervariable V3-V4 region of the 16S rRNA gene, with primers 341F and 806R [31, 32]. The fungal diversity was analyzed using the primers ITS1 and ITS2 according to [5]. Library preparations for Illumina sequencing were performed with a double PCR-step approach, according to Gobbi et al. [32] for 16S rRNA gene and Gobbi et al. [7] for ITS. Sequencing was performed on Illumina’s MiSeq platform using the V2 500 cycles reagent kit.

### Bioinformatics and statistical analysis

Illumina reads were demultiplexed using bcl2fastq V.2.17.1.14 (Illumina). Adapters were trimmed with Trim Galore v0.4 https://github.com/FelixKrueger/TrimGalore.git running cutadapt v1.8.3 [33] and primer sequences were deleted from the 5’ ends of each read using the custom script (https://github.com/padbr/asat/blob/master/strip_degen_primer.py). Trimmed reads were processed according to the Uparse pipeline [34] using Usearch v.10.0.240 i86linux64 with the following differences: i) quality filtering and trimming were performed using usearch–fastq_filter–maxee 1.0 [35], ii) merged reads for 16S rRNA which are outside of the range of 373 bp to 453 bp were discarded and iii) Operational Taxonomic Unit (OTU) clustering was performed using Unoise3. OTU tables were built using the otutab function of Usearch. Samples with less than 5000 reads mapped onto OTUs were discarded. Taxonomy assignment was done using the sintax function [36] of Usearch with the Ribosomal Database Project (RDP) reference 16S Training Set v.16 for prokaryotes and UNITE v7.2 Version 01.12.2017 for fungi. The OTUs assigned to chloroplasts and mitochondria were filtered out, after which, OTUs having less than 0.5% relative abundance in at least one sample were rejected. The OTU trees were built using the cluster_agg [37] function of Usearch.

The Rhea pipeline [38] was used to characterize and compare the microbial communities, and is the source of α-diversity measures, non-metric multidimensional scaling (nMDS) clustering based on generalized UniFrac distances (β-diversity), statistical comparisons of OTU abundances and diversity, and non-parametric correlation analyses. The generalized UniFrac distance was preferred to the Bray-Curtis for its consideration of the genetic distance between the community members (OTUs) in each sample with the members in the other samples. Additionally, the generalized Unifrac has the power to detect abundance changes in moderately abundant lineages, and detect those which are rare and/or highly abundant. The balanced Unifrac version, which was proposed by Chen et al., 2012, is helpful in stabilizing distances between samples as the sampling depth increases, and is thus less likely to miss out important data than its other more commonly used Unifrac distance counterparts.

Statistical significance was defined as p ≤ 0.05 and only significant p values are shown. Additionally, the data was analyzed with Qiime 2 2017.9 [39] and PAST v.3 [40]. To determine the significant presence of structures or groupings in the beta diversity data, the nonparametric PERMANOVA test [41] was used, with 999 permutations. To relate environmental variables with the relative abundance of identified species, a Canonical Correspondence Analysis (CCA) [42] was used. For the analysis of the microbial profile, the α community richness and diversity (Shannon index) were analyzed using the non-parametric Kruskal-Wallis test for three or more group comparisons, and the *post hoc* pairwise comparisons test using the nonparametric Mann–Whitney with Bonferroni adjustment to evaluate differences between two groups. To determine the significance of the division into groups of multivariate analyses, a PERMANOVA analysis was implemented [38, 41] with the VEGAN:: Adonis package in R. Raw sequencing data associated with this work was uploaded to the SRA under the BioProject accession number PRJNA640285.

## Results

### Vineyard climatic and soil physicochemical characteristics

The Ullum Valley is located in the Southern Central region of San Juan Province, Argentina, at 550 to 1300 m above sea level. This Valley has a desert climate, with remarkable daily and annual thermal amplitude and low rainfall. Artificial irrigation is a common practice in vineyards, mainly by canals. The average temperature for the three months prior to harvest (registered by the Estación Experimental Agropecuaria–INTA San Juan), showed a slight increase over the years: 26.2°C for 2015, 26.3°C for 2016, and 26.6°C for 2017. Meanwhile, precipitation was lower in 2015, increased in 2016 and slightly decreased in 2017 (S2 Fig). The sampled sites all had loam soils (18–45% sand, 34–59% silt and 18–28% clay). The soil pH was slightly higher in samples from FN than in those from FA, although all in the range from 7.9 to 8.6, corresponding to mild-moderate alkaline soils. Further details of soil physicochemical characteristics are shown in the S1 Table.

For a better visualization of differences in soil physicochemical characteristics among vintages and vineyards, a principal component analysis (PCA) was performed (S3 Fig). The PCA analysis showed a clear distinction between FN and FA vineyards in the content of organic matter, carbon, nitrogen, available phosphorus and clay. FN samples were more affected by pH and sand content, while available phosphorus, clay, lime and organic nutrients had a higher influence on FA samples. The phosphorus content was clearly high in FA samples of 2015 (FAMA15) and PCA analysis showed clearly the effects on samples from this site. A Mann Whitney U test, however, showed no significant differences between the soil physical and chemical analysis of analyzed vineyards.

### OTU data coverage

The results obtained are based on a dataset consisting of 36 samples and two marker genes, the V3-V4 hypervariable region of the 16S rRNA gene and the fungal ITS1, both sequenced with Illumina technology. The current dataset was employed to analyze the microbiome associated with the rhizospheres of MA and CS cultivars, for three consecutive years (vintages 2015, 2016 and 2017). For each gene marker 36 soil samples were initially analysed, out of which only 34 were successfully processed for fungal community and 29 for bacterial community. The fungal community dataset comprised 2,751,340 quality-filtered reads, with 19,107 to 202,851 reads per sample, while bacterial community dataset included 537,607 quality-filtered reads, with 5,924 to 64,257 reads per sample (S2 Table). The rarefaction analysis was applied to visualize each gene marker dataset, showing the saturation depth of samples (S4 Fig). For both prokaryotic and fungal communities, the rarefaction curves indicated less than expected coverage since not all samples reached the cut-off value.

### Fungal and bacterial rhizospheric diversity

**Fig 1:**
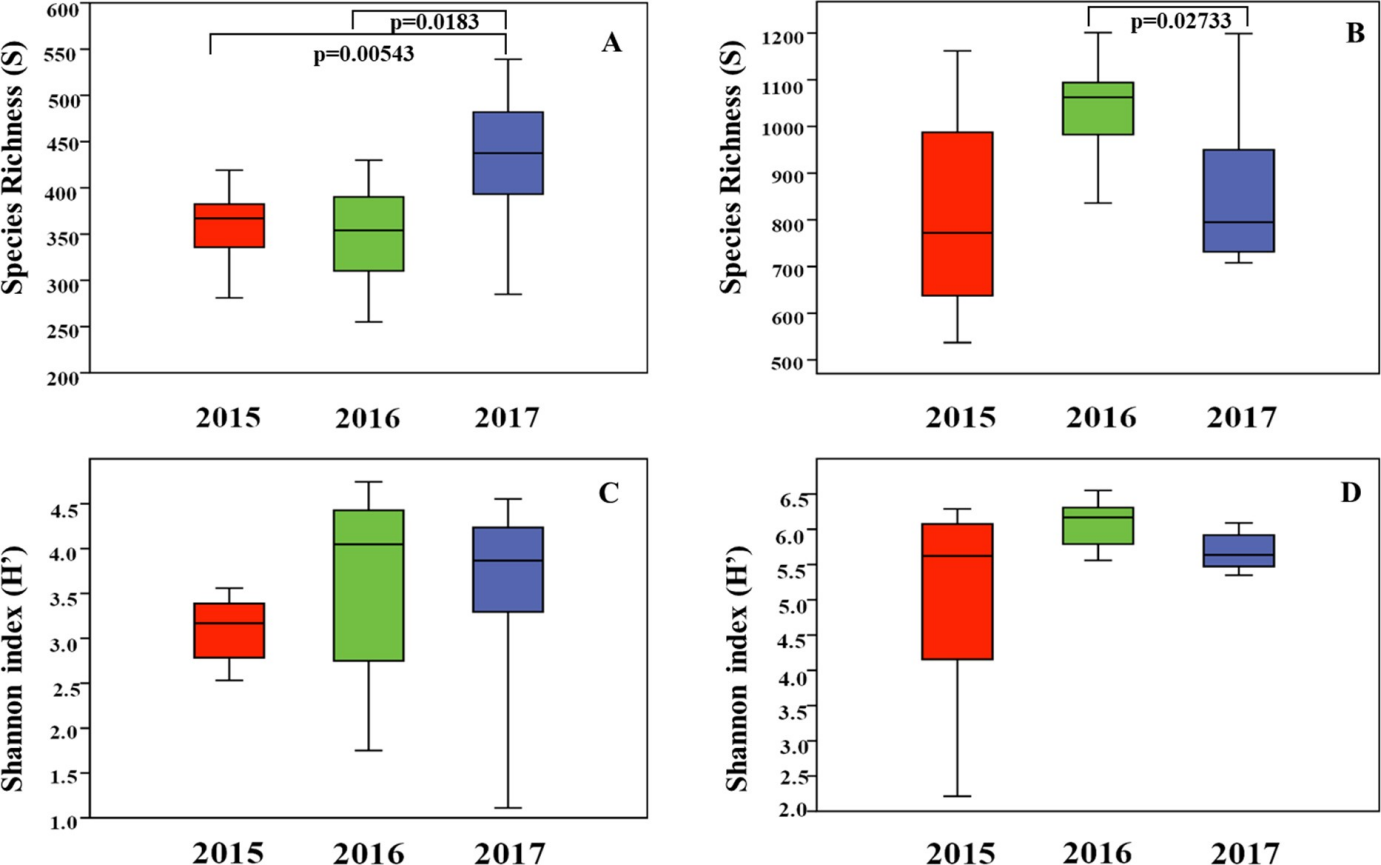
Vintage boxplot of microbial species Richness (S) and Shannon Index (H’) values. Boxplots (A) and (B) represent the fungal and prokaryotic species richness variations, respectively. Correspondingly, figures (C) and (D) represent the fungal and prokaryotic Shannon Index estimate variations. *Pairwise-comparisons* between vintages for (S) and (H’) in both communities are also established. The statistical analysis was done separately for each community that included an initial (S) and (H’) comparison between all three vintages using a Kruskal Wallis test (Richness: p = 0.0024 for fungi and p = 0.0142 for prokaryotes; Shannon Index: p = 0.0323 for prokaryotes). The identified pairwise-comparisons results were obtained through a *post hoc* Mann–Whitney Bonferroni adjusted test.

Two estimators of α-diversity, species (OTU) richness and Shannon’s Index, were used. The average richness per sample ranged from 298 to 447 for fungi, and from 641 to 1,117 for bacteria, while the average Shannon Index per sample varied between 2.53 and 4.35 for fungi and between 3.88 and 6.36 for bacteria (S3 Table). To evaluate the vintage effect on annual rhizosphere microbial composition, the same diversity indexes were plotted by year of sampling (Fig 1). The highest number of OTUs in the fungal and bacterial communities were found in 2017 and 2016, respectively. The species richness comparison between all three vintages using the non-parametric Kruskal-Wallis test showed significant differences in both fungal and bacterial communities (Kruskal-Wallis p = 0.0024 and p = 0.0142, respectively). The Mann-Whitney Bonferroni adjusted pairwise comparison tests showed that fungal species richness significantly differed between 2015 and 2017 (p = 0.00543) and between 2016 and 2017 (p = 0.0183), but not between 2015 and 2016 (Fig 1A). The same approach, applied to bacterial communities, showed differences in species richness only between 2016 and 2017 (p = 0.02733) (Fig 1B). Additionally, the Shannon Index did not show any important variation for fungal and bacterial communities in the pairwise comparisons between vintages (Fig 1C and 1D). However, a significant difference was observed in the bacterial communities when all three year samples were considered (Kruskal-Wallis p = 0.0323). Additional supplementary material with the data point values used in the statistical analysis of Species Richness and Shannon diversity is provided in S4 Table.

**Fig 2:**
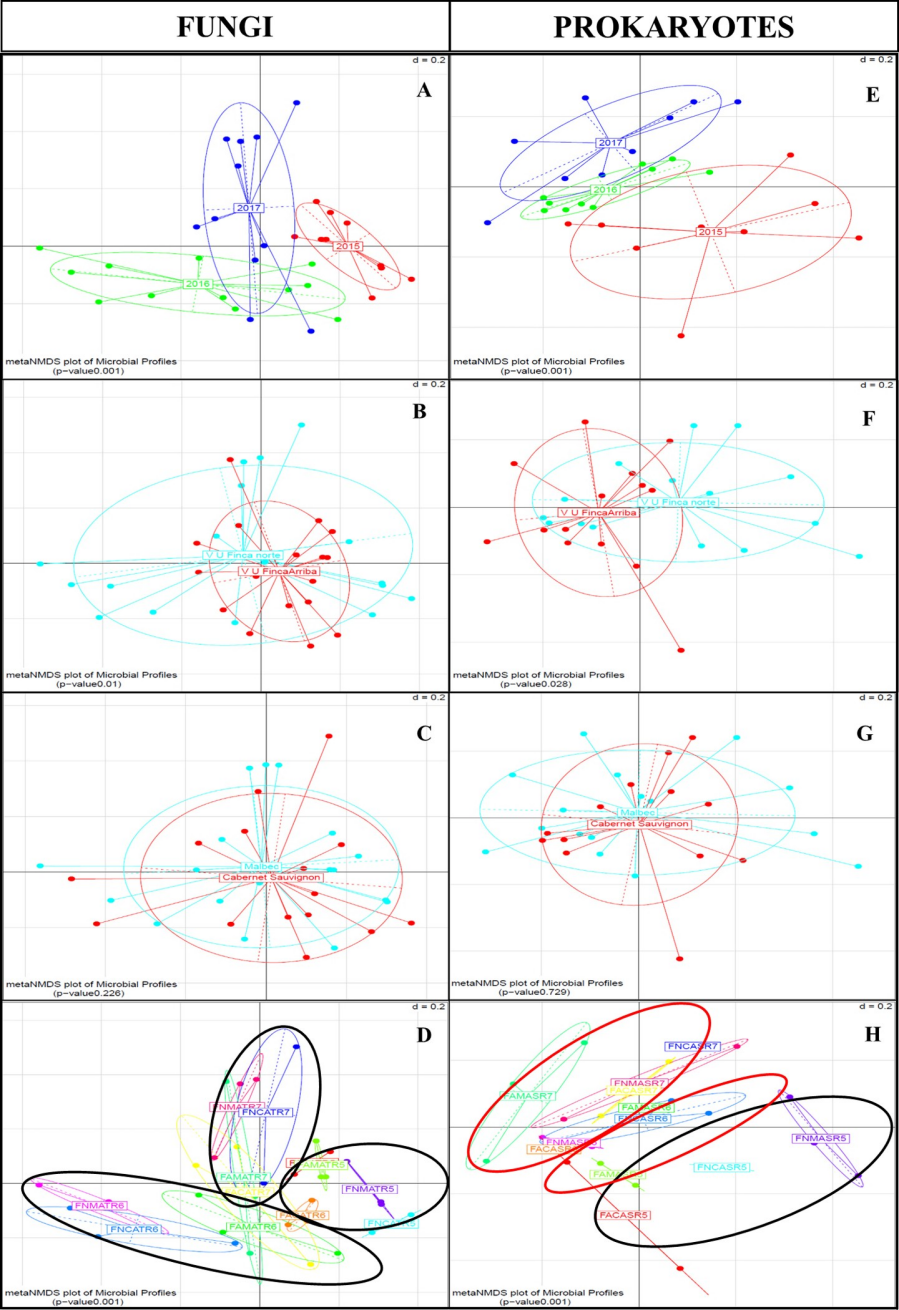
Non-metric Multidimensional Scaling (NMDS) of microbial communities by vintage, location, cultivar and plots. (A) Fungal communities grouped according to the vintage, (B) location, (C) cultivar and (D) plot. (E) Prokaryotic communities grouped according to the vintage, (F) location, (G) cultivar and (D) plot. Samples were grouped at 20% of dissimilarity distance using the generalized Unifrac metric. P values to observe significant difference between communities were obtained through a PERMANOVA test, and are represented on the left hand corner of each figure.

To better estimate microbial community differences between different rhizosphere populations in the sampled vineyards, the beta diversity was assessed using generalized UniFrac distances [43]. Distances were then visualized using (n)MDS plots, which illustrates differences in rhizosphere associated microbiomes related to vintage and vineyard location, both in fungal (Fig 2A and 2B) and bacterial communities (Fig 2E and 2F), but not linked to grapevine cultivars (Fig 2C and 2G). Differences in the structure of microbial communities in relation to each variable were evaluated through a PERMANOVA test. Vintage year (fungal and bacterial communities p <0.001 values) and vineyard location (fungal community p <0.01, bacterial community p = 0.028) were shown to significantly affect the community compositions. Whereas no significant effect was found for the grapevine cultivar (p = 0.226 and p value = 0.729 for the fungal and bacterial communities, respectively). The Unifrac distance matrix input tables used for the ITS1 and 16s rRNA marker gene analyses, have been provided in S5–S7 Tables according to vintage, cultivar and site.

### Fungal and bacterial taxonomic identification

**Fig 3:**
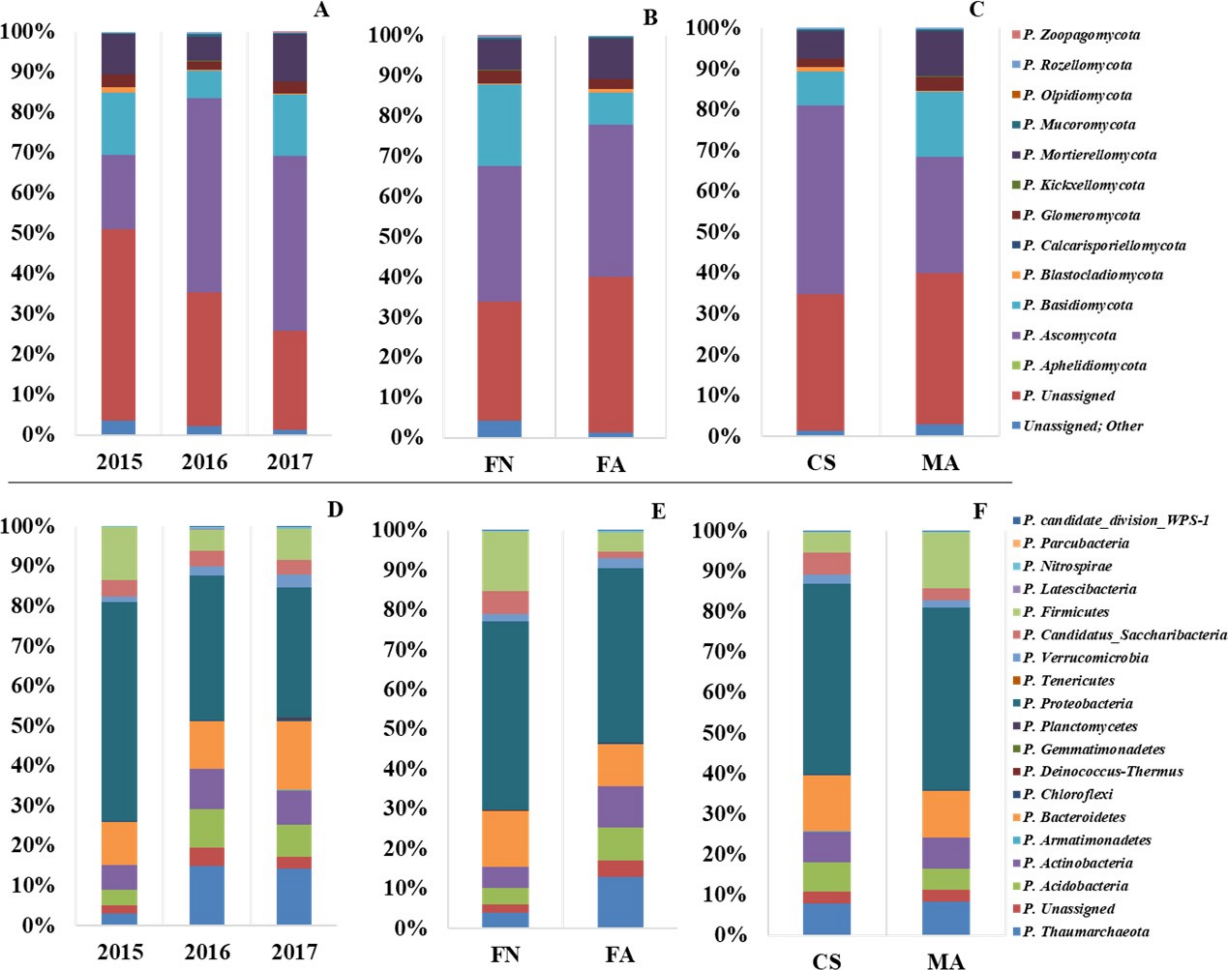
Average relative abundance of the most dominant microbial communities according to the vintage, vineyard location and cultivar. Identified fungal communities classified by (A) vintage year, (B) vineyard location and by (C) cultivar. Identified prokaryotic communities’ classified by (D) vintage year (E) vineyard location and (F) cultivar. Only the communities with abundance > 0.1% are represented.

From taxonomic analyses we observed 12 classified phyla for fungal rhizosphere-associated communities, where the dominant group of assigned OTUs was Ascomycota, with an abundance of 47% for vintage 2016 (Fig 3A). Other important groups were Basidiomycota (2015: 15.3%; 2016: 6.8%; 2017: 15.0%) and Mortierellomycota (2015: 9.9%; 2016: 5.7%; 2017: 11.6%). However, surprisingly, Glomeromycota (an arbuscular mycorrhizal fungi), was found at a low yet noticeable amount (2–3%). The comparison between vineyards (Fig 3B) showed that the relative abundance of Basidiomycota was higher in FN than in FA rhizosphere (20% in FN and 8% in FA). The relative abundances of the remaining phyla did not show great differences between vineyards. Fig 3C shows that in the Cabernet Sauvignon cultivar, Ascomycota was the predominant phylum, with a relative abundance of 48%, compared to 23% in Malbec cultivar. On the other hand, the relative abundance of Basidiomycota was higher in rhizosphere of Malbec cultivar (16%), compared to Cabernet Sauvignon cultivar (8%). S5A Fig shows differences in taxonomic analysis classified by sample. Supplementary OTU abundances for both gene markers are provided in S8 Table.

At the order level, the analysis showed that the abundance of *Dothideomycetes* Clade (22%) is coincident with a high proportion of fungi from *Pleosporales* Order (21%). Members of the *Pleosporales* order showed a significant increase between 2015 (5%) and 2016 (36.8%), largely explaining the increase of Ascomycota in that same period. Their relative abundance was higher in Cabernet Sauvignon cultivar (29.8%) and FA location (24%), while in FN location and Malbec cultivar the values were 15% and 13.6%, respectively. Other orders with minor presence in the fungal communities were: *Hypocreales* (7%), *Agaricales* (7%) and *Helotiales* (2.1%) from the Ascomycota group, *Mortierellales* (9%) from the Mortierellomycota group, and *Glomerales* (1.9%) from the Glomeromycota group. Ascomycota phyla were represented by the genera *Fusarium*, *Lophiostoma*, *Metarhizium* and *Mycoarthris* (relative abundance > 0.5%). S9 Table provides the additional abundance and taxonomic classification data of the top 10 fungal genera identified for each cultivar.

With the aim of investigating the presence of taxonomic groups specific to vintage year, vineyard or grapevine cultivar, the identified taxa were compared at genus level (S6 Fig). The data revealed that 102 of 206 identified genera were present in all three vintage years (S6A Fig), 151 of 206 were shared between vineyards (S6B Fig), and 169 of 206 were shared between grapevine cultivars (S6C Fig). This analysis only considered presence/absence of genera, without accounting for the proportion of these genera in samples. When vintage year and vineyards were compared, only the 2015 year and Finca Norte (FN) samples showed a 1% of exclusive genera in bacterial communities, while in fungal communities the exclusive genera did not exceed 0.1%, according to wine cultivar.

The dominant phyla in the rhizosphere prokaryotic communities were: *Proteobacteria*, *Bacteroidetes*, *Firmicutes*, *Actinobacteria*, and *Acidobacteria*, as well as the archaeal group *Thaumarchaeota* (Fig 3). The less abundant phyla found included *Chloroflexi*, *Verrucomicrobia*, *Parcubacteria*, *Candidatus division* WPS-1, *Candidatus*_*Saccharibacteria*, and *Nitrospirae*. On the other hand, the groups *Armatimonadetes*, *Deinococcus-Thermus*, *Gemmatimonadetes*, *Planctomycetes*, *Tenericutes*, *Latescibacteria*, and *Parcubacteria* were only present in some of the sampled sites (S5B Fig). Annual variations in bacterial community structure showed a noticeable decrease in *Proteobacteria* (55% in 2015 to 36% in 2016) and *Firmicutes* (13.3% in 2015 to 5.3% in 2016). The archaeal group showed a significant increase in relative abundance between 2015 and 2016 (3% to 15%). Between 2016 and 2017 the more evident changes were those corresponding to *Bacteroidetes* (11.8% in 2016 to 17.3% in 2017) and *Firmicutes* (5.3% in 2016 to 7.9% in 2017) (Fig 3D).

The *Proteobacteria* decrease, according to vintage year, was explicable by reductions of *Rhodospirillales* belonging to the α-*Proteobacteria*, and *Enterobacteriales* and *Pseudomonadales*, belonging to the γ-*proteobacteria* (S7 Fig). The more abundant bacterial classes found (75% of OTUs) corresponded to *Alphaproteobacteria* (25%), *Nitrososphaerales* (10.9%), *Gammaproteobacteria* (10.9%), *Cytophagia* (8.1%), *Actinobacteria* (7.7%), *Bacilli* (7.1%) and *Sphingobacteria* (6.3%). For Archaea, the most relatively abundant genus was *Nitrososphaera*.

Regarding the distribution of bacterial phyla in vineyards (Fig 3E), *Firmicutes* was found in higher proportion in FN than in FA (15% and 5%, respectively). Conversely, other phyla were observed in higher relative abundance in FA than in FN: *Actinobacteria* (10 and 5%, respectively), *Acidobacteria* (8 and 4%, respectively), and *Thaumarchaeota* (13 and 4%, respectively). Additionally, the relative abundance of *Firmicutes* was three times higher in FN than in FA (15 and 4.8%, respectively). Differences in the community microbial structures, according to grapevine cultivar, revealed that *Firmicutes* were found in lower relative abundance in CS (5%) than in MA (14%). Meanwhile, *Candidatus Saccharibacteria* showed an inverse pattern, with a higher abundance in CS (5.2%) than in MA (20.85%) (Fig 3F).

To analyze the presence of bacterial taxonomic groups specific to a vintage year, vineyard or cultivar, the taxa assigned at the genus level were compared. It was found that of the 214 identified genera, 148 were present in the three vintages, 177 in both vineyards and 182 in both cultivars (S6D–S6F Fig). In these comparisons, the proportion of exclusive genera among years, cultivar and vineyard did not exceed 0.3% of the relative abundance of each group.

**Fig 4:**
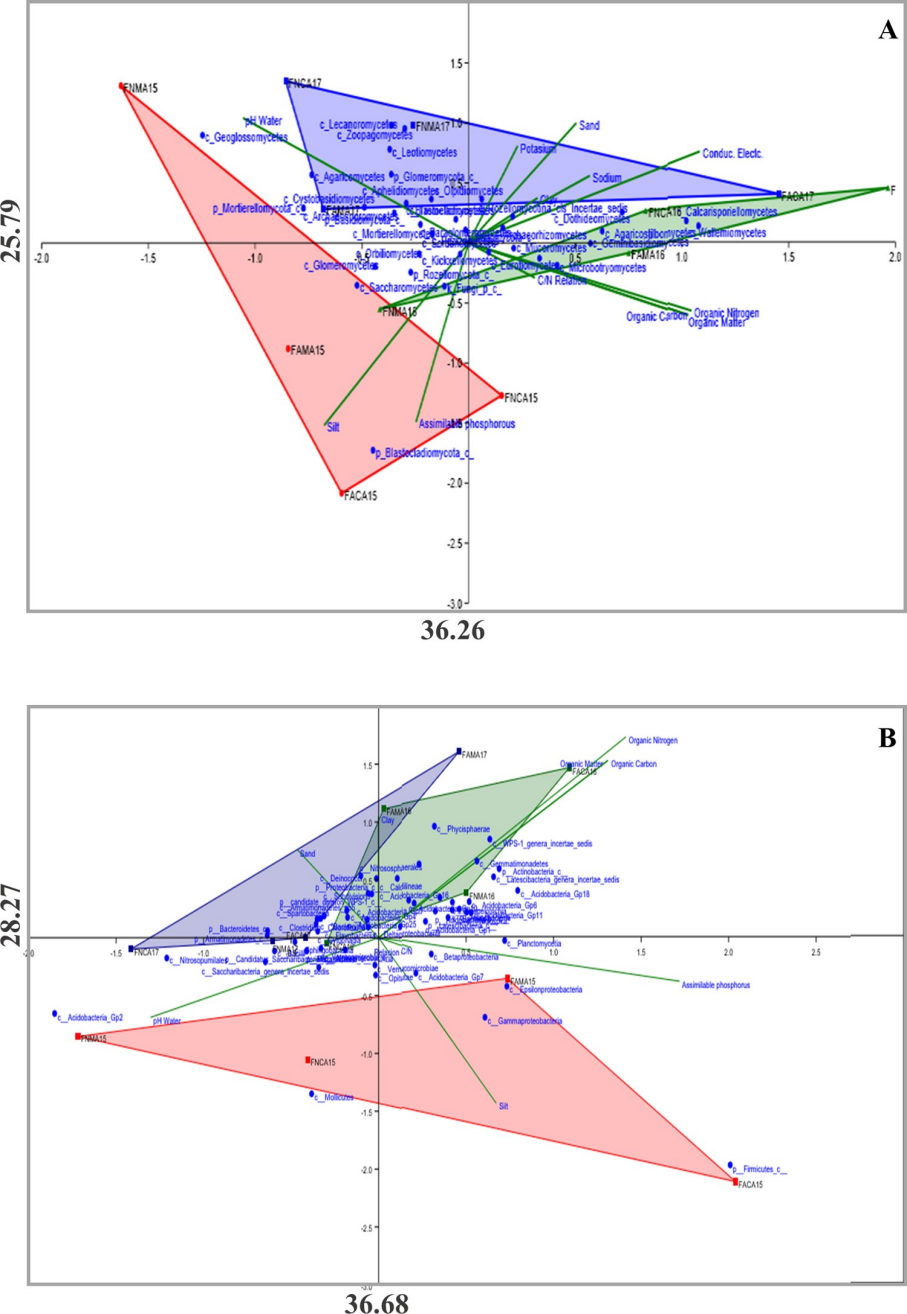
Canonical Correspondence Analysis (CCA) of fungal and bacterial communities. (A) Represents the CCA analysis of the main abundant fungal classes and (B) represents the CCA analysis of the main abundant bacterial classes classified. The 2015 samples are marked in red, the 2016 in green and the 2017 in blue.

To investigate the influence of different soil parameters on the composition of bacterial and fungal communities, a CCA analyses was performed (Fig 4). These analyses showed a 63.93% of variability for fungal communities (Fig 4A), and a 61.98% for bacterial communities (Fig 4B). In the figures, a sample separation may be observed according to vintage year. The position of FAMA15 and FACA15 was strongly influenced by the phosphorus available content in soil. The same behavior was observed in β- γ- ε-*Proteobacteria*, an unassigned class of *Firmicutes*, and *Acidobacteria Gp7*. Most bacteria were found in soils with the highest content of organic matter, nitrogen and carbon, with lower pH samples.

Regarding the distribution of the fungal communities, one group was located in sites with higher contents of organic matter, carbon, nitrogen, and greater abundances of sand and clay (*Dothideomycetes*, *Eurotiomycetes*, *Agaricostilbomycetes*, *Calcarisporiellomycetes*, *Geminibasidiomycetes*, *Microbotryomycetes*, *Wallemiomycetes*). The *Saccharomycetes*, *Glomeromycetes*, *Rozellomycotina* and an unassigned class of *Blastocladiomycetes* were associated with soils with higher contents of available phosphorus and silt. In contrast, *Geoglossomycetes*, *Agaricomycetes* and unassigned groups, belonging to *Basidiomycota* and *Mortierellomycota*, were associated with soils with higher pH.

The relative abundance of bacterial genera capable of solubilizing phosphorus reached, for *Pseudomonas*, 3.2% in 2015, 0.2% in 2016, and 0.4% in 2017. For *Pantoea*, values were 0.44% in 2015, 0.022% in 2016, and 0.007% in 2017. A similar observation was done when vineyards were compared (FA: *Pseudomonas* 3.7%, *Pantoea* 0.5%, and FN: *Pseudomonas* 0.5%, *Pantoea* 0.02%). For fungal genera, a noticeable variation in the relative abundance of *Glomerales* was found related to vintage year, but not to vineyard (2.9% in 2015, 1.3% in 2016, and 1.5% in 2017).

## Discussion

To our knowledge, this work is the first to study the ITS1 marker gene in rhizosphere soil of Malbec grapevines and the first to study fungal and bacterial grapevine-associated rhizosphere communities along three different vintages, in vineyards from Argentina. The analysis included initial evaluation of the α and β diversities, as well as the effects of different soil components on microbial community structure and dynamics. In a previous study utilizing the DGGE technique, Vega-Avila et al. [14] compared the bacterial microbiome associated to rhizosphere of grapevines grown under conventional and organic practices, also in vineyards from the San Juan Province. They found that differences observed between bacterial communities were poorly explained by variations in the physicochemical properties of the rhizosphere. Zarraonaindia et al. [3] characterized the bacterial communities associated with Merlot grapevines, using 16S rRNA amplicon sequencing and shotgun metagenomics, to unravel the influences of host cultivar, soil edaphic parameters, and grapevine developmental stage on its composition, structure, and function, analyzing three grapevine parts (roots, leaves, and grapes or flowers) and the associated soil. They found that microbial communities of soil and roots (rhizosphere) were significantly influenced by soil pH and C: N ratio. The pH was previously identified as an important variable in relation to the structure of soil-associated microbiome [44]. This research confirmed the influence of some soil properties on the structure of microbial communities. We observe that both, pH and nutrient content of soil (organic carbon and nitrogen, C/N, phosphorous), are key factors modifying the composition of fungal and bacterial communities of a vineyard.

On the other hand, we also evaluate the importance of soil available phosphorus. Phosphorus is the second most important soil macronutrient, after nitrogen, and it is found mainly in insoluble form, which makes difficult for plants to absorb it [45–47]. However, fungal and bacterial communities associated with vineyard soils are able to transform the insoluble phosphorous into a form absorbable by plants [48]. We found that available phosphorous was particularly high in 2015 vintage for FA vineyard. A possible explanation could be found in the abundance of some *Gammaproteobacteria* members, particularly the genera *Pseudomonas* and *Pantoea*. Both genera exhibited an evident phosphate-solubilization activity, capacity mentioned by other authors [49–52]. In the case of fungal communities, the AMF *Glomerales* showed variations relative to vintage year (higher in 2015), but not to vineyard site. This taxa is known as an important nutrient transporter in plants, and is particularly good for acquisition of soil phosphorus [53]. The abundance of other phosphorus-transforming fungal genera, such as *Fusarium*, *Aspergillus*, and *Alternaria* [47], did not exhibit changes relative to vintage year or vineyard site.

According to the nMDS analysis, fungal and bacterial communities were shown to be significantly different and clustered together according to vintage year and vineyard location, but not to vine cultivar. Chou et al. [54] showed that the soil-associated bacterial and fungal microbiome may undergo shifts according to under-vine management (e.g., herbicide application, soil cultivation, and natural vegetation). Although the influence of vineyard management was not evaluated, the similarly implemented agricultural practices on both vineyards, could have influenced the results achieved by overshadowing the wine cultivar effect on the microbial composition of the rhizosphere-associated communities.

Regarding the fungal groups, the major identified populations were *Ascomycetes*, *Basidiomycetes*, and *Mortierellomycetes*. These results agree with findings by Deacon et al. [55], who observed that *Ascomycetes* were typically predominant in agricultural soils while *Basidiomycetes* were most predominant in pastures. Soil fungi could be classified into functional groups, including biological controllers, ecosystem regulators, organic matter decomposer, and composite transformers [56]. *Ascomycetes* play fundamental roles in most soil ecosystems, participating in the decomposition of organic matter, such as dead leaves, stems, and fallen trees [56]. Despite the important roles played by fungi in maintaining soil fertility diversity studies of vine-associated fungal communities have been focused on plant aerial organs [9, 57–59]. Phyllosphere studies have found that *Basidiomycetes* can predominate on leaves, while fermentative *Ascomycete* genera (*Hanseniaspora*, *Candida*, *Metschnikowia* and spoilage yeasts as *Aerobasiidium pullulans*) can predominate on grape cuticles [16]. Recently, Singh et al. [60] showed that *Aureobasidium*, *Cladosporium* and *Alternaria* were the predominant fungal groups on different grape varieties. Chou et al. [54] found that in soil, more abundant genera were *Verticillium*, *Nectria*, *Mortierella*, *Gibberella*, and *Fusarium*, whose abundances depend on soil management. External factors, such as chemical and biological treatments, may also contribute to the composition of vineyard-associated bacterial and fungal communities. However, as Perazzolli et al. [61] showed, the indigenous phyllosphere-associated communities are more dependent upon the effects of vineyard biotic and environmental factors, and can be resilient to various management practices. This could explain why our results showed many core fungal shared genera in samples of both cultivars, locations and vintages (S6 Fig). As previously mentioned, the association of AMF with plants is an important factor for agriculture, since they facilitate the absorption of different mineral nutrients from soil in exchange for fixed carbon provided by plants [62]. Their diversity is mainly influenced by type of soil, rather than by characteristics of plant host or agricultural practices [63–65]. The primers used in this work do not efficiently amplify *Glomeromycetes* [66] and as a consequence there is the possibility of an underestimation of this group (abundance value found 4%) that is so relevant to the structure of fungal communities associated with vineyards [67].

Regarding bacterial communities, taxonomic analyses showed *Proteobacteria* (41%) as the dominant phylum. A study of rhizobacteria associated with field-grown lettuce revealed *Proteobacteria* as the dominant group in different soil types (alluvial loam, diluvial sand and loess loam), with different clades correlating better with a specific soil type [68]. *Proteobacteria* has been commonly identified as the most abundant phylum in crops and could be used as an indicator of soil nutrient richness [69]. Other less abundant phyla were *Bacteroidetes*, *Actinobacteria*, *Firmicutes*, and *Acidobacteria*, showing notable differences in abundances compared to other works. Zarraonaindia et al. [3], analyzing the grapevine rhizosphere associated microbiomes, also observed a predominance of sequences attributable to *Proteobacteria*, followed by *Bacteroidetes*, *Acidobacteria*, *Verrucomicrobia*, and *Actinobacteria*. Later, Novello et al. [70] in a Pinot Noir vineyard of Piedmont (Italy), found higher presence of *Actinobacteria*, *Proteobacteria*, *Gemmatimonadetes*, and *Bacteroidetes*. Vega-Avila et al. [14], studying the diversity of microorganisms associated with Syrah vines from San Juan Province, found *Proteobacteria* as the most abundant phylum, followed by *Bacteroidetes* in conventionally treated vineyards. The organic vineyards, however, had a higher presence of *Firmicutes*, *Acidobacteria*, *Verrucomicrobia* and *Planctomycetes*. In our work, the higher abundances of *Proteobacteria* and *Bacteroidetes* could be related more to the applied agricultural method, while the shifts in their abundances could be more specific to the particular annual demands of each vineyard condition and location in which those treatments are applied.

The genera belonging to these phyla were identified at an abundance >0.6%, being *Rhizobium* genus (2.3%) the most abundant. Although cover crops were not commonly used in the vineyards sampled, it is important to mention that species belonging to *Rhizobium* genus have the capacity to form an endosymbiotic nitrogen-fixing association with legume roots and to promote growth in non-legume plants [71, 72].

The microbiome is clearly an important factor in viticulture and enology, due to roles played by microorganisms in chemical and nutritional properties of vineyard soils. In addition, the microbiome contributes to the grapevine health and yield and also plays a role in the wine fermentation process. Furthermore, several studies indicate that vineyard-associated microbiomes could be susceptible to change in relation to soil type and agricultural practices [73, 74].

The present study showed that the vintage was a relevant variable in affecting the microbial community compositions of both sampled locations. In the wine industry the vintage is also used as a reference, to which the unique annual vineyard conditions influence grape chemistry and can denote unique wine flavors [75]. According to Bokulich et al., [10] vintage can also play an important role on the microbial biogeography of wine grapes and possibly wine itself. Regardless of this effect, the annual variations of the rhizosphere microbiome is still underrated and further efforts to understand how the varying outcomes can affect the yearly wine production quality should be addressed. The impact of soil physicochemical parameters also proved to have an effect on the structure of the rhizosphere-associated microbiota, as well as the vineyard environment characteristics and the biological aspect inherent to the wine cultivar.

## Conclusion

Our results have shown that bacterial and fungal communities present in rhizosphere soils are primarily affected by the conditions of the soil composition, which plays a direct role in the stabilization of the microbial populations. Despite the fact that we did not evaluate all possible changing environmental and grapevine management effects on the vineyards, we observed significant annual shifts in the microbial populations attributed to the changing content of soil components, especially in absorbable phosphorus. We observed no significant differences between cultivar microbial communities yet we did observe annual shifts that changed between locations. This does not indicate that the cultivar has no role in rhizosphere microbial selection or composition, but instead it infers that other external factors, such as agricultural management, may be more relevant to this effect. Collectively, our results provide a first overview of the microbial abundance patterns present in San Juan vineyards. Additionally, given the commonly agronomic practices adopted in this province, the outcomes of our study may open the door to more sustainable vineyard management practices by using alternative agricultural methods.

## Supporting information

S1 Fig Vineyard sample-taking pattern, field-taking procedures and pooling of biological replicate samples. (A) Samples were taken at a 7 m distance into the vineyard and away from the edge of each sampling plot. (B) The sample-taking pattern included 9 plants at a 2.5 m distance between each vine, contained within a 14 m^2^ quadrant plot. Samples were pooled and integrated into three composite biological replicates (the marked symbols indicate how biological triplicate samples included rhizosphere soil from each row). (C) Finally, the rhizosphere was taken from grapevine roots at a 20 cm distance from the vine trunk and 30 cm deep into the soil. * The first layer of surface soil was discarded prior to taking the actual study root samples.
(PDF)

S2 Fig San Juan province climatic data records prior to the sample-taking vintages. This graph includes the average temperatures and precipitation data registered in both San Juan INTA-Stations (Pocitos and Aero), considering only the three and a half months prior to the sample-taking of the 2015, 2016 and 2017 vintages.
(PDF)

S3 Fig Principal Component Analysis (PCA) of the soils’ physical and chemical properties. Soil samples clearly grouped separately between vineyard locations. The red circle contains the Finca Arriba (FA) samples that seemed to be mostly influenced by the water pH and sand content, while the Finca Norte (FN) samples circled in blue, seemed to be mainly influenced by the sampled organic components, available phosphorus and, lime and sand content.
(PDF)

S4 Fig Rarefaction curves of rhizosphere ITS1 and 16s rRNA gene markers’ saturation depth analyses. The rarefaction analysis showed the gene marker dataset saturation depth of both (A) fungal and (B) prokaryotic rhizosphere soil communities. The curves indicated less than expected coverage since not all samples reached the cut-off value.
(PDF)

S5 Fig Average relative abundance of the dominant communities classified according to each sample. (A) Identified fungal communities classified by sample and (B) identified prokaryotic communities classified by sample.
(PDF)

S6 Fig Venn diagram indicating the assigned shared fungal genera. Shared microbial populations of identified fungal genera classified according to (A) vintage, (B) vineyard location and (C) cultivar. Shared microbial populations of identified prokaryote genera classified according to (D) vintage, (E) vineyard location and (F) cultivar. The overlapping areas indicate the number of shared genera.
(PDF)

S7 Fig *Proteobacteria* relative abundance classified according to sampling year. Only the orders with a relative abundance >1% are represented.
(PDF)

S1 Table Physicochemical parameters of vineyard rhizospheric soils. *FN: Finca Norte; FA: Finca Arriba; MA: Malbec; CA: Cabernet Sauvignon; 15, 16 and 17 stand for the vintages 2015, 2016 and 2017.
(PDF)

S2 Table ITS1 and 16s marker gene dataset count summary statistics. (PDF)

S3 Table α-diversity analysis including Species (OTU) Richness and Shannon’s Index for prokaryotic and fungal gene markers, 16s rRNA and ITS1, respectively. Numbers with an (*) indicate the highest and the lowest average indexes per group of replicates for each sample. The table contains the average diversity results of all sample triplicates along with their respective standard deviation.
(PDF)

S4 Table ITS1 and 16s rRNA gene marker vintage data point values per sample, used for the Species Richness and Shannon Index α-diversity statistical analyses. (PDF)

S5 Table ITS1 and 16s rRNA marker genes Unifrac distance matrixes input table according to vintage. (XLSX)

S6 Table ITS1 and 16s rRNA marker genes Unifrac distance matrixes input table according to cultivar. (XLSX)

S7 Table ITS1 and 16s rRNA marker genes Unifrac distance matrixes input table according to site or parcel. (XLSX)

S8 Table ITS1 and 16s rRNA marker genes’ OTU list and abundance classified by sample. (XLSX)

S9 Table Top 10 fungal abundance and taxonomically identified genera. (PDF)

## References

[1] Gupta V, Bramley R, Greenfield P, Yu J, Herderich MJ (2019) Vineyard soil microbiome composition related to Rotundone concentration in Australian cool climate 'peppery' Shiraz grapes. Frontiers in microbiology 10: 1607 10.3389/fmicb.2019.01607 31379773 PMC6646731

[2] Gilbert JA, van der Lelie D, Zarraonaindia I (2014) Microbial terroir for wine grapes. Proc. Natl. Acad. Sci. U.S.A 111: 5–6. 10.1073/pnas.1320471110 24309378 PMC3890852

[3] Zarraonaindia I, Owens SM, Weisenhorn P, West K, Hampton-Marcell J, Lax S, et al (2015) The soil microbiome influences grapevine-associated microbiota. mBio (online) 6 e02527e02514. 10.1128/mBio.02527-14 25805735 PMC4453523

[4] Pieterse CMJ, de Jonge R, Berendsen RL (2016) The soil-borne supremacy. Trends Plant Sci. 21: 171–173. 10.1016/j.tplants.2016.01.018 26853594

[5] Del Frari G, Gobbi A, Aggerbeck MR, Oliveira H, Hansen LH, Ferreira RB (2019a) Characterization of the wood mycobiome of *Vitis vinifera* in a vineyard affected by Esca. spatial distribution of fungal communities and their putative relation with leaf symptoms. Frontiers in plant science 10: 910 10.3389/fpls.2019.01405 31354777 PMC6640213

[6] Del Frari G, Gobbi A, Aggerbeck MR, Oliveira H, Hansen LH, Ferreira RB (2019b) Fungicides and the grapevine wood mycobiome: A case study on Tracheomycotic Ascomycete *Phaeomoniella chlamydospora* reveals potential for two novel control strategies. Frontiers in Plant Science 10: 1405 10.3389/fpls.2019.01405 31737020 PMC6836639

[7] Gobbi A, Kyrkou I, Filippi E, et al (2020) Seasonal epiphytic microbial dynamics on grapevine leaves under biocontrol and copper fungicide treatments. Sci Rep 10: 681 10.1038/s41598-019-56741-z 31959791 PMC6971271

[8] Wei Y, Wu Y, Yan Y, Zou W, Xue J, Ma W, et al (2018) High-throughput sequencing of microbial community diversity in soil, grapes, leaves, grape juice and wine of grapevine from China. PLoS One 13:e0193097 10.1371/journal.pone.0193097 29565999 PMC5863948

[9] Singh P, Santoni S, This P, Peros JP (2018) Genotype-environment interaction shapes the microbial assemblage in grapevine's phyllosphere and carposphere: An NGS Approach. Microorganisms 6 10.3390/microorganisms6040096 30248973 PMC6313654

[10] Bokulich NA, Thorngate JH, Richardson PM, Mills DA (2014) Microbial biogeography of wine grapes is conditioned by cultivar, vintage, and climate. Proc Natl Acad Sci U.S.A 111: 139–148. 10.1073/pnas.1317377110 24277822 PMC3890796

[11] Knight S, Klaere S, Fedrizzi B, Goddard MR (2015) Regional microbial signatures positively correlate with differential wine phenotypes: Evidence for a microbial aspect to terroir. Scientific Reports 5: 1–10. 10.1038/srep14233 26400688 PMC4585847

[12] Bokulich NA, Collins TS, Masarweh C, Allen G, Heymann H, Ebeler SE, et al (2016). Associations among wine grape microbiome, metabolome, and fermentation behavior suggest microbial contribution to regional wine characteristics. mBio 7: e00631–16. 10.1128/mBio.00631-16 27302757 PMC4959672

[13] Belda I, Navascués E, Marquina D, Santos A, Calderón F, Benito S (2016) Outlining the influence of non-conventional yeasts in wine ageing over lees. Yeast 33: 329–338. 10.1002/yea.3165 27017923

[14] Vega-Avila AD, Gumiere T, Andrade PAM, Lima-Perim JE, Durrer A, Baigori M, et al (2014) Bacterial communities in the rhizosphere of *Vitis vinifera L*. cultivated under distinct agricultural practices in Argentina. Antonie van Leeuwenhoek 107: 575–588. 10.1007/s10482-014-0353-7 25527391

[15] Marasco R, Rolli E, Fusi M, Michoud G & Daffonchio D (2018) Grapevine rootstocks shape underground bacterial microbiome and networking but not potential functionality. Microbiome 6: 3 10.1186/s40168-017-0391-2 29298729 PMC5751889

[16] Barata A, Malfeito-Ferreira M, Loureiro V (2012) The microbial ecology of wine grape berries. International Journal of Food Microbiology 153: 243–259. 10.1016/j.ijfoodmicro.2011.11.025 22189021

[17] Belda I, Zarraonaindia I, Perisin M, Palacios A and Acedo A (2017) From Vineyard Soil to Wine Fermentation: Microbiome Approximations to explain the “terroir” Concept. Front. Microbiol. 8: 821 10.3389/fmicb.2017.00821 28533770 PMC5420814

[18] Roca, P. (2019). International Organization of Vine and Wine (OIV). In State of the Vitiviniculture World Market (p. PowerPoint.PDF). Geneva, Switzerland.

[19] INV. (2018). INFORME ANUAL DE SUPERFICIE 2018. Mendoza.

[20] Koorem K, Gazol A, Öpik M, Moora M, Saks Ü, et al (2014) Soil nutrient content influences the abundance of soil microbes but not plant biomass at the small-scale. PLoS ONE 9(3): e91998 10.1371/journal.pone.0091998 24637633 PMC3956881

[21] Jacoby R, Peukert M, Succurro A, Koprivova A, Kopriva S (2017) The role of soil microorganisms in plant mineral nutrition—current knowledge and future directions. Front. Plant Sci. 8:1617 10.3389/fpls.2017.01617 28974956 PMC5610682

[22] Bulgari D, Casati P, Crepaldi P, Daffonchio D, Quaglino F, et al (2011) Restructuring of endophytic bacterial communities in grapevine yellows-diseased and recovered *Vitis vinifera L*. plants. Appl Environ Microb 77: 5018–5022. 10.1128/AEM.00051-11 21622794 PMC3147392

[23] Campisano A, Antonielli L, Pancher M, Yousaf S, Pindo M, et al (2014) Bacterial endophytic communities in the grapevine depend on pest management. PLoS ONE 9(11): e112763 10.1371/journal.pone.0112763 25387008 PMC4227848

[24] Belda I, Gobbi A, Ruiz J, Celis M, Ortiz-Alvarez R, Acedo A, et al (2020) Microbiomics to define wine *terroir*. Elsevier Reference Module in Food Sciences, 1–14. 10.1016/B978-0-08-100596-5.22875-8

[25] Marban L (2005) Acondicionamiento de la muestra de suelo previo al ensayo. Algunas consideraciones. ed. Marbán L y Ratto S. AACS. II (2): 65–67.

[26] Walkley A, Black A (1934) An examination of Degtareff method for determining soil organic matter and proposed modification of the chromic acid tritation method. Soil Sci. 37:39–38.

[27] Davies BE (1974) Loss-on-ignition as an estimate of soil organic matter. Soil. Sci. Soc. Amer. Proc., 38, 150–151. 10.1111/j.1365-2389.1964.tb00247.x

[28] Bremner JM (1960) Determination of nitrogen in soil by the Kjeldahl method. 10.1017/S0021859600021572

[29] Bray RH, Kurtz LT (1945) Determination of total, organic and available forms of phophorus in soils. Soil Sci. 59: 39–45.

[30] Kilmer V, Alexander L (1949) Methods of making mechanical analysis of soil. Soils Sci. 68: 15–24.

[31] Takahashi S, Tomita J, Nishioka K, Hisada T, Nishijima M (2014) Development of a prokaryotic universal primer for simultaneous analysis of bacteria and archaea using next-generation sequencing. PLoS One 9 (8): e105592 10.1371/journal.pone.0105592 25144201 PMC4140814

[32] Gobbi A, Santini RG, Filippi E, Ellegaard-Jensen L, Jacobsen CS, Hansen L (2018) Quantitative and qualitative evaluation of the impact of the G2 enhancer, bead sizes and lysing tubes on the bacterial community composition during DNA extraction from recalcitrant soil core samples based on community sequencing and qPCR. PLoS ONE 14(4): e0200979 10.1371/journal.pone.0200979 30973938 PMC6459482

[33] Martin M (2011) Cutadapt removes adapter sequences from high-throughput sequencing reads. 2011 17:3 10.14806/ej.17.1.200

[34] Edgar RC (2013) UPARSE: highly accurate OTU sequences from microbial amplicon reads. Nat Methods 10: 996–998. 10.1038/nmeth.2604 23955772

[35] Edgar RC, Flyvbjerg H (2015) Error filtering, pair assembly and error correction for next-generation sequencing reads. Bioinformatics 31: 3476–3482. 10.1093/bioinformatics/btv401 26139637

[36] Edgar RC (2016) SINTAX: a simple non-Bayesian taxonomy classifier for 16S and ITS sequences. bioRxiv 074161. 10.1101/074161

[37] Edgar RC (2010) Search and clustering orders of magnitude faster than BLAST. Bioinformatics 26: 2460–2461. 10.1093/bioinformatics/btq461 20709691

[38] Lagkouvardos I, Fischer S, Kumar N & Clavel T (2017) Rhea: a transparent and modular R pipeline for microbial profiling based on 16S rRNA gene amplicons. PeerJ 5: e2836 10.7717/peerj.2836 28097056 PMC5234437

[39] Caporaso JG, Kuczynski J, Stombaugh J, et al (2010) QIIME allows analysis of high-throughput community sequencing data. Nat Methods 7: 335–336. PMCID: PMC3156573 10.1038/nmeth.f.303 20383131 PMC3156573

[40] Hammer Ø, Harper DAT & Ryan PD (2001) Past: Paleontological statistics software package for education and data analysis. Palaeontologia Electronica 4(1): 1–9.

[41] Anderson MJ (2001) A new method for non‐parametric multivariate analysis of variance. Austral Ecology 26: 32–46. 10.1111/j.1442-9993.2001.01070.pp.x

[42] Ter Braak CJF (1986) Canonical Correspondence Analysis: A New Eigenvector Technique for Multivariate Direct Gradient Analysis. Ecology 67: 1167–1179. 10.2307/1938672

[43] Chen J, Bittinger K, Charlson ES, Hoffmann C, Lewis J, Wu GD (2012) Associating microbiome composition with environmental covariates using generalized UniFrac distances. Bioinformatics 28: 2106–2113. 10.1093/bioinformatics/bts342 PMCID: PMC3413390 22711789 PMC3413390

[44] Steenwerth KL, Drenovsky RE, Lambert JJ, Kluepfel DA, Scow KM & Smart DR (2008) Soil morphology, depth and grapevine root frequency influence microbial communities in a Pinot noir vineyard. Soil Biology and Biochemistry 40: 1330–1340. 10.1016/j.soilbio.2007.04.031

[45] Azzis G, Bajsa N, Haghjou T, Taulé C, Valverde A, Igual J, et al (2012) Abundance, diversity and prospecting of culturable phosphate solubilizing bacteria on soils under crop–pasture rotations in a no-tillage regime in Uruguay. Appl. Soil Ecol. 61 320–326.

[46] Tak HI, Ahmad F, Babalola OO, Inam A (2012) Growth, photosynthesis and yield of chickpea as influenced by urban wastewater and different levels of phosphorus. Int. J. Plant Res. 2 6–13. 10.5923/j.plant.20120202.02

[47] Alori ET, Glick BR, Babalola OO (2017) Microbial phosphorus solubilization and its potential for use in sustainable agriculture. Frontiers in microbiology 8: 971 10.3389/fmicb.2017.00971 28626450 PMC5454063

[48] Zuccaro A. (2019). Plant phosphate status drives host microbial preferences: a trade‐off between fungi and bacteria. The EMBO journal. e104144 10.15252/embj.2019104144 31886558 PMC6960440

[49] Son HJ, Park GT, Cha MS & Heo MS (2006) Solubilization of insoluble inorganic phosphates by a novel salt- and pH-tolerant *Pantoea agglomerans* R-42 isolated from soybean rhizosphere. Bioresource technology 97: 204–210. 10.1016/j.biortech.2005.02.021 16171676

[50] Castagno LN, Estrella MJ, Sannazzaro AI, Grassano AE & Ruiz OA (2011) Phosphate-solubilization mechanism and in vitro plant growth promotion activity mediated by Pantoea eucalypti isolated from Lotus tenuis rhizosphere in the Salado River Basin (Argentina). J Appl Microbiol 110: 1151–1165. 10.1111/j.1365-2672.2011.04968.x 21299771

[51] Oteino N, Lally RD, Kiwanuka S, Lloyd A, Ryan D, Germaine KJ, et al (2015) Plant growth promotion induced by phosphate solubilizing endophytic Pseudomonas isolates. Front Microbiol 6: 745 10.3389/fmicb.2015.00745 PMCID: PMC4510416 26257721 PMC4510416

[52] Linu MS, Asok AK, Thampi M, Sreekumar J & Jisha MS (2019) Plant Growth Promoting Traits of Indigenous Phosphate Solubilizing Pseudomonas aeruginosa Isolates from Chilli (Capsicumannuum L.) Rhizosphere. Communications in Soil Science and Plant Analysis 50: 444–457. 10.1080/00103624.2019.1566469

[53] Müller LM, Harrison MJ (2019) Phytohormones, miRNAs, and peptide signals integrate plant phosphorus status with arbuscular mycorrhizal symbiosis. Current Opinion in Plant Biology, 50:132–139. 10.1016/j.pbi.2019.05.004 31212139

[54] Chou MY, Vanden Heuvel J, Bell TH, Panke-Buisse K & Kao-Kniffin J (2018) Vineyard under-vine floor management alters soil microbial composition, while the fruit microbiome shows no corresponding shifts. Sci Rep 8: 11039 10.1038/s41598-018-29346-1 30038291 PMC6056419

[55] Deacon LJ, Pryce-miller EJ, Frankland JC, Bainbridge BW, Moore PD, Robinson CH (2006) Diversity and function of decomposer fungi from a grassland soil. Soil Biology and Biochemistry 38: 7–20. 10.1016/j.soilbio.2005.04.013

[56] Frac M, Hannula SE, Belka M & Jedryczka M (2018) Fungal biodiversity and their role in soil health. Front Microbiol 9: 707 PMCID: PMC5932366 10.3389/fmicb.2018.00707 29755421 PMC5932366

[57] Setati ME, Jacobson D, Andong UC, Bauer FF (2012) The vineyard yeast microbiome, a mixed model microbial map. PLoS ONE 7: e52609 10.1371/journal.pone.0052609 23300721 PMC3530458

[58] Pinto C, Pinho D, Sousa S, Pinheiro M, Egas C, Gomes AC (2014) Unravelling the diversity of grapevine microbiome. PLoS One 9: e85622 10.1371/journal.pone.0085622 PMCID: PMC3894198 24454903 PMC3894198

[59] Setati ME, Jacobson D, Bauer FF (2015) Sequence-based analysis of the Vitis vinifera L. cv Cabernet Sauvignon grape must mycobiome in three South African vineyards employing distinct agronomic systems. Front Microbiol 6: 1358 10.3389/fmicb.2015.01358 PMCID: PMC4663253 26648930 PMC4663253

[60] Singh P, Santoni S, Weber A, This P, Peros JP (2019) Understanding the phyllosphere microbiome assemblage in grape species (Vitaceae) with amplicon sequence data structures. Sci Rep 9: 14294 10.1038/s41598-019-50839-0 31586145 PMC6778096

[61] Perazzolli M, Antonielli L, Storari M, Puopolo G, Pancher M, Giovannini O, et al (2014) Resilience of the natural phyllosphere microbiota of the grapevine to chemical and biological pesticides. *A*pplied and Environmental Microbiology 80: 3585–3596. 10.1128/AEM.00415-14 24682305 PMC4054146

[62] Smith SE, Read D (2008) Mycorrhizal symbiosis (3rd ed.). Academic Press.

[63] Schreiner RP, Mihara KL (2009) The diversity of arbuscular mycorrhizal fungi amplified from grapevine roots (Vitis vinifera L.) in Oregon vineyards is seasonally stable and influenced by soil and vine age. Mycologia 101: 599–611. 10.3852/08-169 19750939

[64] Balestrini R, Magurno F, Walker C, Lumini E, Bianciotto V (2010) Cohorts of arbuscular mycorrhizal fungi (AMF) in Vitis vinifera, a typical Mediterranean fruit crop. Environmental Microbiology Reports 2: 594–604. 10.1111/j.1758-2229.2010.00160.x 23766230

[65] Likar M, Hančević K, Radić T, Regvar M (2013) Distribution and diversity of arbuscular mycorrhizal fungi in grapevines from production vineyards along the eastern Adriatic coast. Mycorrhiza 23: 209–219. 10.1007/s00572-012-0463-x 23053577

[66] Krüger M, Stockinger H, Krüger C, Schüßler A (2009) DNA-based species level detection of Glomeromycota: one PCR primer set for all arbuscular mycorrhizal fungi. New Phytologist 183: 212–223. 10.1111/j.1469-8137.2009.02835.x 19368665

[67] Trouvelot S, Bonneau L, Redecker D, van Tuinen D, Adrian M, Wipf D (2015) Arbuscular mycorrhiza symbiosis in viticulture: a review. Agron. Sustain. Dev. 35:1449–1467. 10.1007/s13593-015-0329-7

[68] Schreiter S, Ding GC, Heuer H, Neumann G, Sandmann M, Grosch R, et al (2014) Effect of the soil type on the microbiome in the rhizosphere of field-grown lettuce. Front Microbiol 5: 144 10.3389/fmicb.2014.00144 24782839 PMC3986527

[69] Beckers B, Op De Beeck M, Weyens N, Boerjan W, Vangronsveld J (2017) Structural variability and niche differentiation in the rhizosphere and endosphere bacterial microbiome of field-grown poplar trees. Microbiome 5: 25 10.1186/s40168-017-0241-2 28231859 PMC5324219

[70] Novello G, Gamalero E, Bona E, Boatti L, Mignone F, Massa N, et al (2017) The rhizosphere bacterial microbiota of Vitis vinifera cv. Pinot Noir in an integrated pest management vineyard. Front Microbiol 8: 1528 10.3389/fmicb.2017.01528 PMCID: PMC5557794 28855895 PMC5557794

[71] Zhang XX, Tang X, Sheirdil RA, Sun L, Ma XT (2014) *Rhizobium rhizoryzae sp*. nov., isolated from rice roots. Int J Syst Evol Microbiol 64: 1373–1377. 10.1099/ijs.0.056325-0 24449787

[72] Bacon CW, White JF (2015) Functions, mechanisms and regulation of endophytic and epiphytic microbial communities of plants. Symbiosis 68: 87–98. 10.1007/s13199-015-0350-2

[73] Burns KN, Kluepfel DA, Strauss SL, Bokulich NA, Cantu D, Steenwerth KL (2015) Vineyard soil bacterial diversity and composition revealed by 16S rRNA genes: differentiation by geographic features. Soil Biology and Biochemistry 91: 232–247. 10.1016/j.soilbio.2015.09.002

[74] Burns KN, Bokulich NA, Cantu D, Greenhut RF, Kluepfel DA, O’Geen AT, et al (2016) Vineyard soil bacterial diversity and composition revealed by16S rRNA genes: differentiation by vineyard management. Soil Biol. Biochem.103: 337–348. 10.1016/j.soilbio.2016.09.007

[75] Mozell MR and Thach L. (2014). The impact of climate change on the global wine industry: Challenges & solutions. Wine Economics and Policy 3 (2014) 81–89. 10.1016/j.wep.2014.08.001

