# Supplementary material for: Influence of vintage, geographic location and cultivar on the structure of microbial communities associated with the grapevine rhizosphere in vineyards of San Juan Province, Argentina
Source: PLoS One. 2020 Dec 14;15(12):e0243848. doi: 10.1371/journal.pone.0243848 (PMC7735631; doi:10.1371/journal.pone.0243848)
Supplement: S1 Fig — (A) Samples were taken at a 7 m distance into the vineyard and away from the edge of each sampling plot. (B) The sample-taking pattern included 9 plants at a 2.5 m distance between each vine, contained within a 14 m2 quadrant plot. Samples were pooled and integrated into three composite biological replicates (the marked symbols indicate how biological triplicate samples included rhizosphere soil from each row). (C) Finally, the rhizosphere was taken from grapevine roots at a 20 cm distance from the vine trunk and 30 cm deep into the soil. * The first layer of surface soil was discarded prior to taking the actual study root samples. (PDF) [file pone.0243848.s001.pdf]

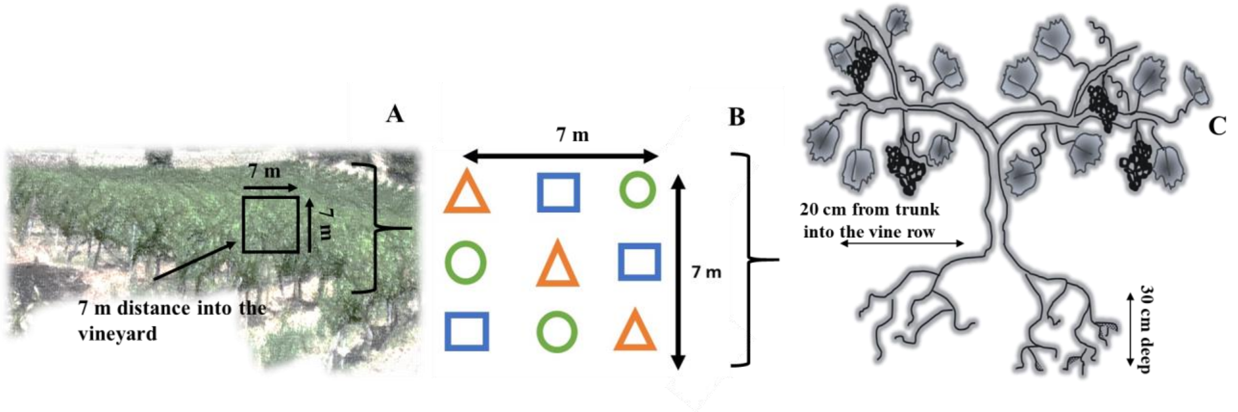

S1 Fig. Vineyard technique for suitable sample selection, field taking procedures and pooling of biological soil sample replicates.
