# Supplementary figures and images for: Influence of vintage, geographic location and cultivar on the structure of microbial communities associated with the grapevine rhizosphere in vineyards of San Juan Province, Argentina
Source: PLoS One. 2020 Dec 14;15(12):e0243848. doi: 10.1371/journal.pone.0243848 (PMC7735631; doi:10.1371/journal.pone.0243848)

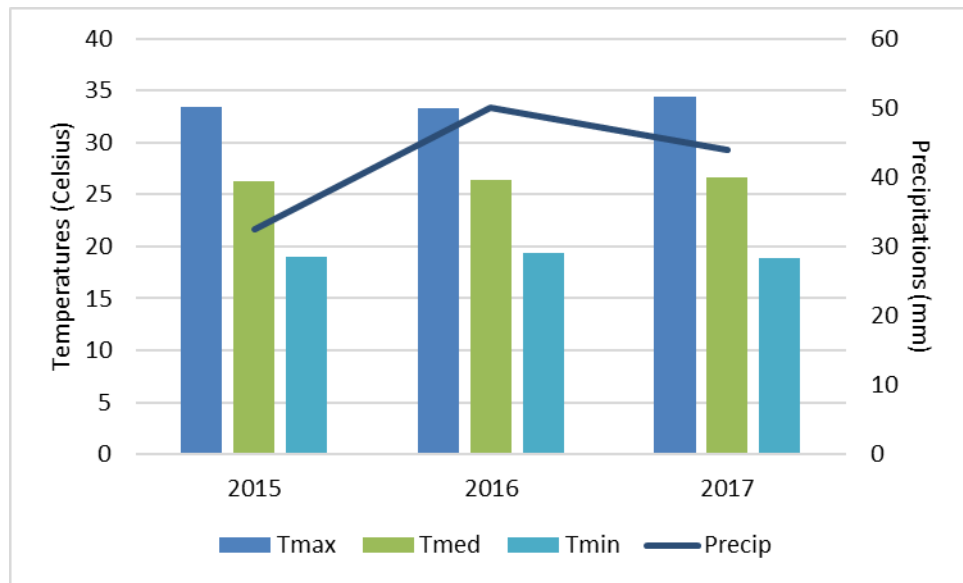

**S2 Fig. San Juan province climatic data records prior to the sample-taking vintages.**

Supplement: S2 Fig — This graph includes the average temperatures and precipitation data registered in both San Juan INTA-Stations (Pocitos and Aero), considering only the three and a half months prior to the sample-taking of the 2015, 2016 and 2017 vintages. (PDF) [file pone.0243848.s002.pdf]

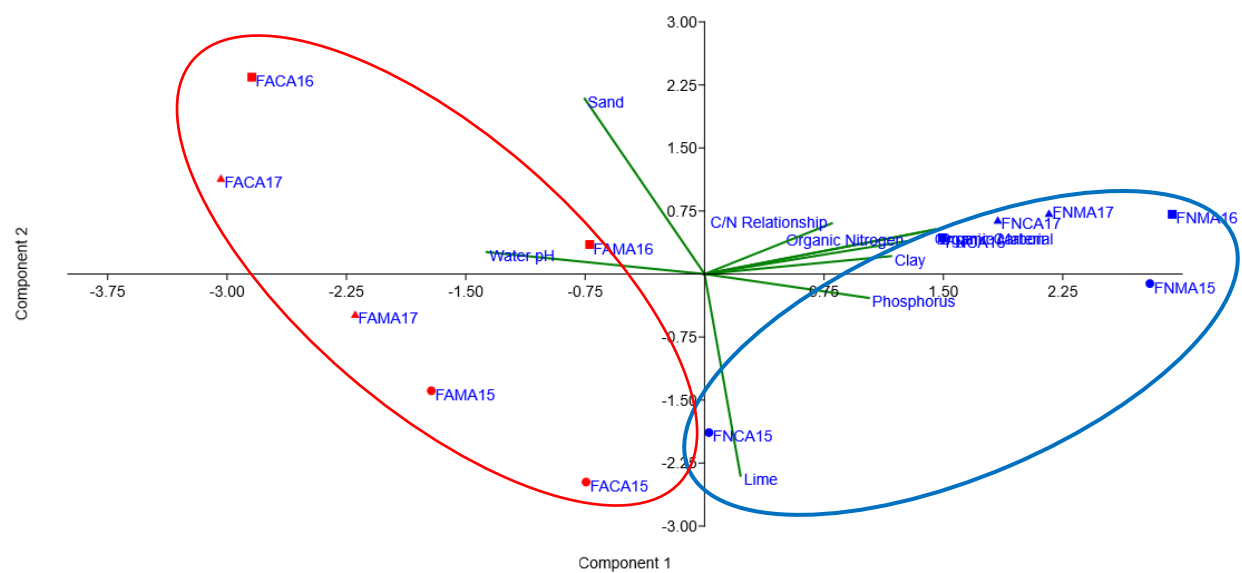

**S3 Fig. Principal Component Analysis (PCA) of the soils' physical and chemical properties.**

Supplement: S3 Fig — Soil samples clearly grouped separately between vineyard locations. The red circle contains the Finca Arriba (FA) samples that seemed to be mostly influenced by the water pH and sand content, while the Finca Norte (FN) samples circled in blue, seemed to be mainly influenced by the sampled organic components, available phosphorus and, lime and sand content. (PDF) [file pone.0243848.s003.pdf]

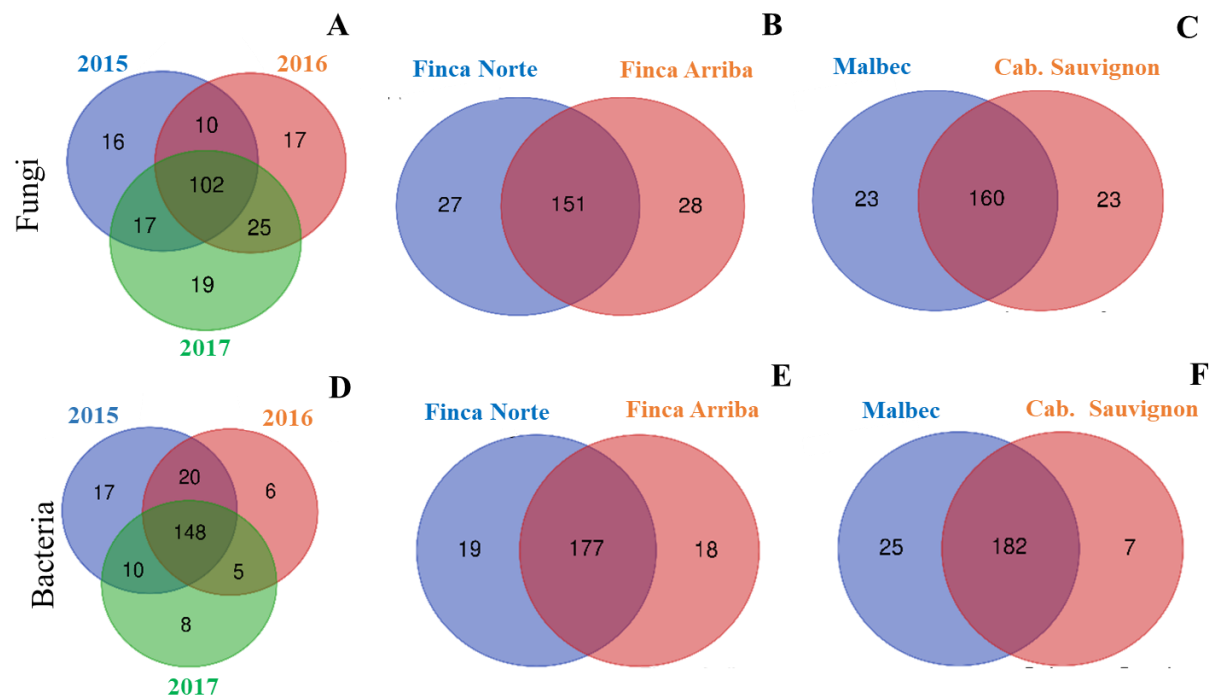

**S6 Fig.** Venn diagram indicating the assigned shared fungal genera.

Supplement: S6 Fig — Shared microbial populations of identified fungal genera classified according to (A) vintage, (B) vineyard location and (C) cultivar. Shared microbial populations of identified prokaryote genera classified according to (D) vintage, (E) vineyard location and (F) cultivar. The overlapping areas indicate the number of shared genera. (PDF) [file pone.0243848.s006.pdf]
