# Supplementary material for: Influence of vintage, geographic location and cultivar on the structure of microbial communities associated with the grapevine rhizosphere in vineyards of San Juan Province, Argentina
Source: PLoS One. 2020 Dec 14;15(12):e0243848. doi: 10.1371/journal.pone.0243848 (PMC7735631; doi:10.1371/journal.pone.0243848)
Supplement: S5 Fig — (A) Identified fungal communities classified by sample and (B) identified prokaryotic communities classified by sample. (PDF) [file pone.0243848.s005.pdf]

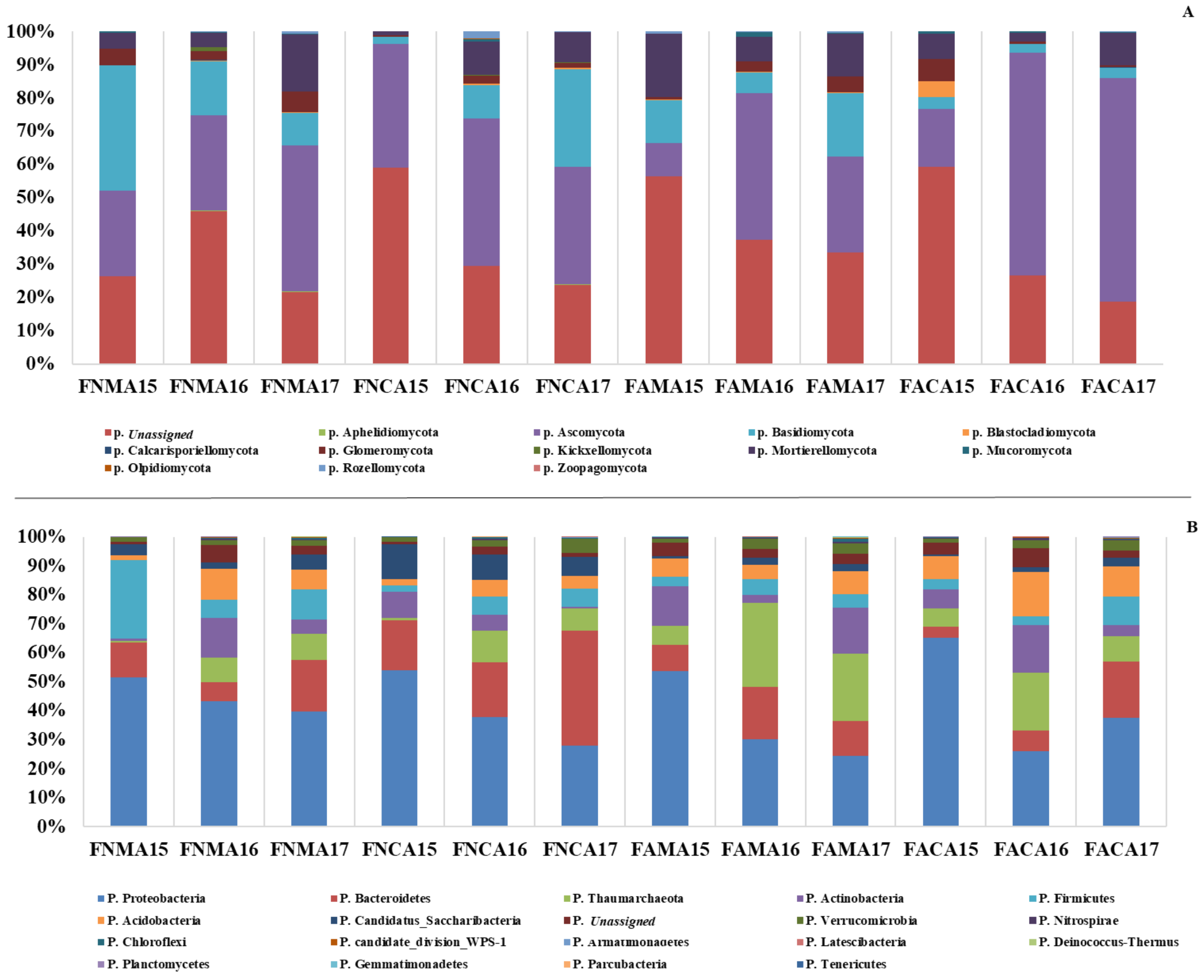

S5 Fig. Average relative abundance of the dominant communities classified according to each sample.
