# Supplementary material for: Influence of vintage, geographic location and cultivar on the structure of microbial communities associated with the grapevine rhizosphere in vineyards of San Juan Province, Argentina
Source: PLoS One. 2020 Dec 14;15(12):e0243848. doi: 10.1371/journal.pone.0243848 (PMC7735631; doi:10.1371/journal.pone.0243848)
Supplement: S7 Fig — Only the orders with a relative abundance >1% are represented. (PDF) [file pone.0243848.s007.pdf]

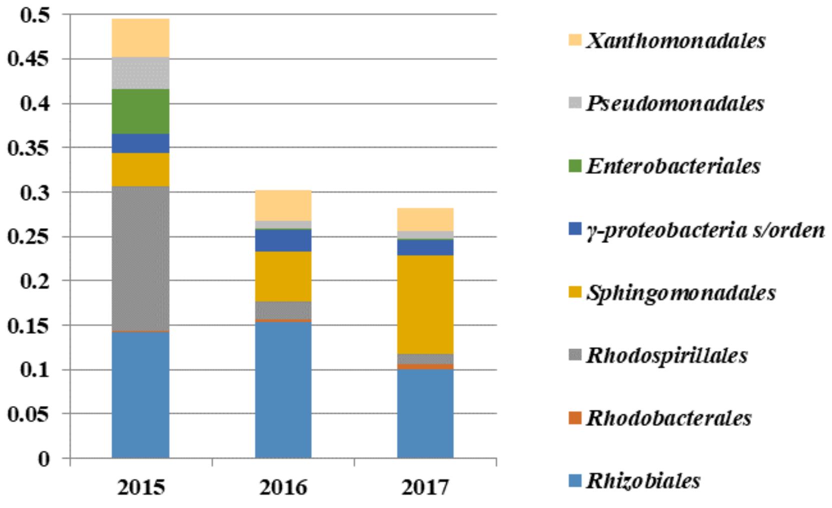

S7 Fig. ***Proteobacteria* relative abundance classified according to sampling year.** Only the orders with a relative abundance >1% are represented.
