# Supplementary material for: Influence of vintage, geographic location and cultivar on the structure of microbial communities associated with the grapevine rhizosphere in vineyards of San Juan Province, Argentina
Source: PLoS One. 2020 Dec 14;15(12):e0243848. doi: 10.1371/journal.pone.0243848 (PMC7735631; doi:10.1371/journal.pone.0243848)
Supplement: S1 Table — *FN: Finca Norte; FA: Finca Arriba; MA: Malbec; CA: Cabernet Sauvignon; 15, 16 and 17 stand for the vintages 2015, 2016 and 2017. (PDF) [file pone.0243848.s008.pdf]

S1 Table. Physicochemical parameters of vineyard rhizospheric soils.

Sample	Clay (% p/p)	Lime (% p/p)	Sand (% p/p)	pH	Organic matter (% p/p)	Organic carbon (% p/p)	Organic nitrogen (% p/p)	C/N ratio	Assimilable Phosphorus mg/Kg
FNMA15	24.80	50.30	21.60	8.33	1.34	0.78	0.08	9.75	5.80
FNCA15	20.10	59.10	18.50	8.16	1.67	0.97	0.10	9.70	8.60
FAMA15	28.00	47.40	23.10	8.07	2.40	1.39	0.12	11.58	71.30
FACA15	17.80	56.90	22.50	8.10	1.81	1.05	0.10	10.50	50.80
FNMA16	20.40	45.40	32.80	8.25	1.97	1.14	0.13	8.77	13.70
FNCA16	18.80	34.40	45.30	8.34	1.41	0.82	0.08	10.25	9.70
FAMA16	25.20	45.80	26.10	7.98	2.84	1.65	0.15	11.00	25.50
FACA16	23.30	45.90	28.00	8.11	2.50	1.45	0.15	9.67	22.30
FNMA17	21.60	45.90	28.80	8.29	1.38	0.80	0.09	8.89	5.90
FNCA17	17.20	41.30	37.10	8.63	1.48	0.86	0.08	10.75	8.50
FAMA17	26.70	43.50	27.60	7.99	2.50	1.45	0.14	10.36	28.90
FACA17	28.40	43.00	26.50	7.92	2.21	1.28	0.11	11.64	24.60

*FN: Finca Norte; FA: Finca Arriba; MA: Malbec; CA: Cabernet Sauvignon; 15, 16 and 17 stand for the vintages 2015, 2016 and 2017.
