# Supplementary material for: Influence of vintage, geographic location and cultivar on the structure of microbial communities associated with the grapevine rhizosphere in vineyards of San Juan Province, Argentina
Source: PLoS One. 2020 Dec 14;15(12):e0243848. doi: 10.1371/journal.pone.0243848 (PMC7735631; doi:10.1371/journal.pone.0243848)
Supplement: S2 Table — (PDF) [file pone.0243848.s009.pdf]

S2 Table. **ITS1 and 16s marker gene dataset count summary statistics.**

	**ITS1**	**16s rRNA**
Number of samples	34	29
Number of observations	2863	2810
Total count	2751340	537607
Table density (fraction of non-zero values)	0.167	0.409
Counts/sample summary:		
Min	19107	5924
Max	212851	64257
Median	82409	13425
Mean	80921	18538
Std. Dev.	39168	12631
