# Supplementary material for: Influence of vintage, geographic location and cultivar on the structure of microbial communities associated with the grapevine rhizosphere in vineyards of San Juan Province, Argentina
Source: PLoS One. 2020 Dec 14;15(12):e0243848. doi: 10.1371/journal.pone.0243848 (PMC7735631; doi:10.1371/journal.pone.0243848)
Supplement: S3 Table — Numbers with an (*) indicate the highest and the lowest average indexes per group of replicates for each sample. The table contains the average diversity results of all sample triplicates along with their respective standard deviation. (PDF) [file pone.0243848.s010.pdf]

S3 Table. **α-diversity analysis including Species (OTU) Richness and Shannon's Index for prokaryotic and fungal gene markers, 16s rRNA and ITS1, respectively.**

Sample	Gene Marker	**Richness (S)**	**Shannon Index**
FNMA15	16s rRNA	641 ± 120*	3.88 ± 1.71*
	ITS1	385 ± 30	3.30 ± 0.22
FNCA15	16s rRNA	782 ± 129	5.72 ± 0.37
	ITS1	298 ± 23*	2.90 ± 0.17
FAMA15	16s rRNA	988 ± 122	5.95 ± 0.31
	ITS1	365 ± 21	2.88 ± 0.41
FACA15	16s rRNA	912 ± 354	5.40 ± 1.25
	ITS1	372 ± 14	3.40 ± 0.24
FNMA16	16s rRNA	1068 ± 50	6.36 ± 0.17
	ITS1	360 ± 25	4.35 ± 0.40*
FNCA16	16s rRNA	1117 ± 73*	5.93 ± 0.19
	ITS1	358 ± 91	3.96 ± 0.61
FAMA16	16s rRNA	907 ± 100	5.63 ± 0.10
	ITS1	333 ± 86	3.78 ± 1.14
FACA16	16s rRNA	1019 ± 49	6.26 ± 0.11
	ITS1	353 ± 28	2.53 ± 0.75*
FNMA17	16s rRNA	978 ± 243	5.69 ± 0.34
	ITS1	447 ± 29*	3.91 ± 0.09
FNCA17	16s rRNA	736	5.58
	ITS1	446 ± 79	3.09 ± 1.45
FAMA17	16s rRNA	771 ± 81	5.58 ± 0.23
	ITS1	429 ± 50	3.99 ± 0.32
FACA17	16s rRNA	839 ± 78	5.84 ± 0.27
	ITS1	419 ± 128	2.97 ± 1.74

Numbers with an (*) indicate the highest and the lowest average indexes per group of replicates for each sample.
