# Supplementary material for: Influence of vintage, geographic location and cultivar on the structure of microbial communities associated with the grapevine rhizosphere in vineyards of San Juan Province, Argentina
Source: PLoS One. 2020 Dec 14;15(12):e0243848. doi: 10.1371/journal.pone.0243848 (PMC7735631; doi:10.1371/journal.pone.0243848)
Supplement: S4 Table — (PDF) [file pone.0243848.s011.pdf]

S4 Table. ITS1 and 16s rRNA gene marker vintage data point values per sample, used for the Species Richness and Shannon Index α-diversity statistical analyses.

	**ITS1**						**16s rRNA**					
	**Species Richness**			**Shannon Index**			**Species Richness**			**Shannon Index**		
	**2015**	**2016**	**2017**	**2015**	**2016**	**2017**	**2015**	**2016**	**2017**	**2015**	**2016**	**2017**
**FNMA**	419	365	479	3.553215	4.744851	4.00355	613	1010	1199	3.79299	6.236401	6.028138
	368	383	441	3.145415	4.353206	3.815066	537	1092	1017	2.213874	6.299945	5.69407
	367	333	422	3.19101	3.947784	3.919275	772	1101	718	5.622892	6.550542	5.348813
**FNCA**	281	428	478	3.023486	4.145771	4.338803		1201	736	5.460644	6.156355	5.579657
	314	392	356	2.78179	4.451199	1.520211	691	1079		5.986171	5.818433	
		255	503		3.282174	3.404879	873	1071			5.822796	
**FAMA**	384	303	483	3.332709	4.163043	4.306481	1074	978	708	6.165001	5.705863	5.498225
	343	267	384	2.531182	2.500414	3.65884	901	836	742	5.730828	5.560693	5.402036
	367	430	421	2.78687	4.669361	4.016117			863			5.840651
**FACA**	362	385	285	3.214825	3.248852	1.110123	1162	1054	928	6.289717	6.337201	6.089443
	382	343	434	3.560349	2.583023	3.259239	662	984	781	4.518843	6.180522	5.561103
		332	539		1.75036	4.553198			809			5.881981
