# Supplementary material for: Influence of vintage, geographic location and cultivar on the structure of microbial communities associated with the grapevine rhizosphere in vineyards of San Juan Province, Argentina
Source: PLoS One. 2020 Dec 14;15(12):e0243848. doi: 10.1371/journal.pone.0243848 (PMC7735631; doi:10.1371/journal.pone.0243848)
Supplement: S9 Table — (PDF) [file pone.0243848.s016.pdf]

S9 Table. Top 10 fungal abundance and taxonomically identified genera.

Taxonomic ID	Malbec	Taxonomic ID	Cab. Sauvignon
p__;c__;o__;f__;g__	0.3661836	p__;c__;o__;f__;g__	0.331309
p__Mortierellomycota;c__Mortierellomycetes;o__Mortierellales;f__Mortierellaceae;g__Mortierella	0.1073933	p__Ascomycota;c__Dothideomycetes;o__Pleosporales;f__;g__	0.258547
p__Ascomycota;c__Dothideomycetes;o__Pleosporales;f__Lophiostomataceae;g__Lophiostoma	0.058539	p__Mortierellomycota;c__Mortierellomycetes;o__Mortierellales;f__Mortierellaceae;g__Mortierella	0.065328
p__Ascomycota;c__Dothideomycetes;o__Pleosporales;f__;g__	0.0550782	p__Basidiomycota;c__Agaricomycetes;o__Agaricales;f__Lycoperdaceae;g__	0.038821
p__Basidiomycota;c__Agaricomycetes;o__Agaricales;f__Agaricaceae;g__Coprinus	0.0422186	p__Ascomycota;c__Sordariomycetes;o__Hypocreales;f__Clavicipitaceae;g__Metarhizium	0.033153
p__Ascomycota;c__Sordariomycetes;o__Hypocreales;f__Nectriaceae;g__Fusarium	0.0400106	p__Ascomycota;c__Sordariomycetes;o__Hypocreales;f__Nectriaceae;g__Fusarium	0.029126
Unassigned;Other;Other;Other;Other;Other	0.0305147	p__Ascomycota;c__Dothideomycetes;o__Pleosporales;f__Lophiostomataceae;g__Lophiostoma	0.024054
p__Basidiomycota;c__Agaricomycetes;o__Auriculariales;f__;g__	0.0240397	p__Ascomycota;c__Sordariomycetes;o__Xylariales;f__;g__	0.016884
p__Glomeromycota;c__Glomeromycetes;o__Glomerales;f__Glomeraceae;g__	0.0191707	Unassigned;Other;Other;Other;Other;Other	0.014713
p__Basidiomycota;c__Agaricomycetes;o__Agaricales;f__Entolomataceae;g__Entoloma	0.0152974	p__Glomeromycota;c__Glomeromycetes;o__Glomerales;f__Glomeraceae;g__	0.014081
