# Supplementary material for: Influence of vintage, geographic location and cultivar on the structure of microbial communities associated with the grapevine rhizosphere in vineyards of San Juan Province, Argentina
Source: PLoS One. 2020 Dec 14;15(12):e0243848. doi: 10.1371/journal.pone.0243848 (PMC7735631; doi:10.1371/journal.pone.0243848)
Supplement: S4 Fig — The rarefaction analysis showed the gene marker dataset saturation depth of both (A) fungal and (B) prokaryotic rhizosphere soil communities. The curves indicated less than expected coverage since not all samples reached the cut-off value. (PDF) [file pone.0243848.s004.pdf]

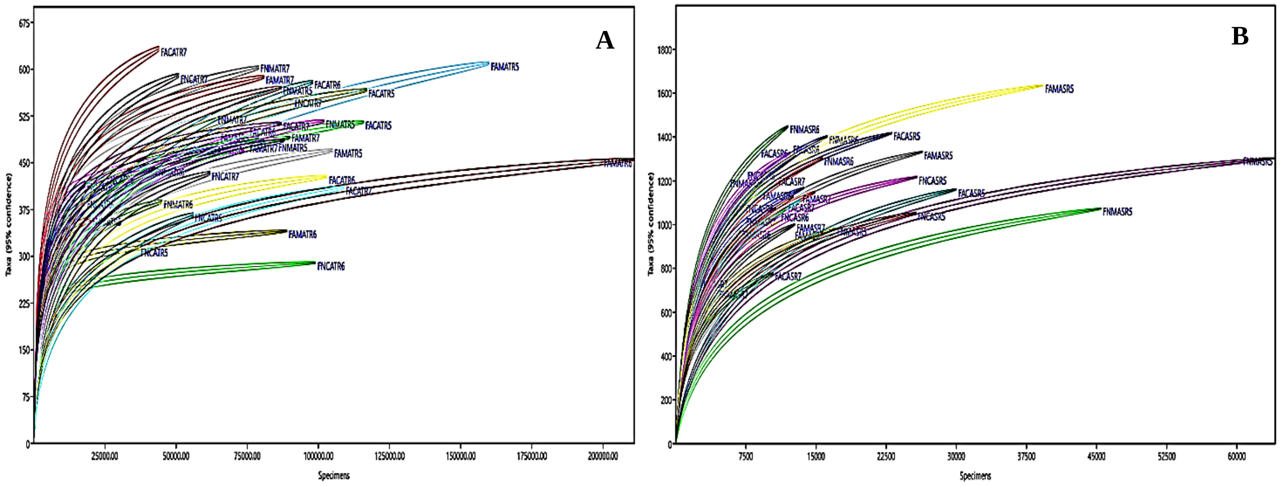


**S4 Fig. Rarefaction curves of rhizosphere ITS1 and 16s rRNA gene markers' saturation depth analyses.**
